# Neurology Undergraduate Medical Education: A Scoping Review

**DOI:** 10.1111/ene.70061

**Published:** 2025-03-13

**Authors:** L. McElligott, A. Ardilouze, J. Moloney, A. ElSheikhId, C. Healy, H. Leahy, K. Babatunde, C. Cahir, P. Murphy, N. Delanty, N. McElvaney, S. Byrne, E. McGovern

**Affiliations:** ^1^ Department of Neurology Beaumont Hospital Dublin Ireland; ^2^ School of Postgraduate Studies Royal College of Surgeons in Ireland Dublin Ireland; ^3^ School of Medicine Royal College of Surgeons in Ireland Dublin Ireland; ^4^ Data Science Centre, School of Population Health Royal College of Surgeons in Ireland Dublin Ireland; ^5^ Royal College of Surgeons in Ireland Library Dublin Ireland; ^6^ FutureNeuro RCSI University of Medicine and Health Sciences Dublin Ireland; ^7^ Children's Health Ireland Crumlin Hospital Dublin Ireland

**Keywords:** education interventions, medical education, neurology

## Abstract

**Purpose:**

To map the current literature on undergraduate neurology medical education and research. Recommendations for future undergraduate neurology education and research are described.

**Method:**

PRISMA—Scoping Review guidelines and Arksey and O′Malley's methodological framework are followed. Four databases and gray literature was searched with Oxford Evidence‐Based Medicine level of evidence applied. A thematic framework was used to identify the main study outcomes. A narrative description and quantitative frequency analysis were used for results.

**Results:**

Nine‐hundred and twenty‐two articles were retrieved, 102 studies met the inclusion criteria. We identified four main study outcomes using a thematic framework. Our review found that (1) the main undergraduate neurology teaching styles are didactic and experiential teaching methods. (2) Research design of undergraduate neurology teaching is heterogenous. (3) The outcome measures most frequently used in undergraduate neurology research are student perception and knowledge.

**Conclusion:**

Undergraduate neurology education research is challenging due to the heterogeneity in research design and teaching methodology. Evidence‐based guidelines are limited. This gap in the literature represents an opportunity to develop tailored guidelines for undergraduate neurology education and research.

## 1 Introduction

Neurological disease is estimated to be one of the leading causes of disability‐adjusted life years and the second leading cause of death worldwide [1]. A growing burden of neurological disease means medical students and doctors are likely to encounter patients with neurological diseases during their training and practice [1, 2].

Neurology is core to undergraduate medical curriculum [1, 3, 16, 24, 161, 167, 170], yet it is considered a difficult subject by medical students [4, 5, 6]. Pre‐clinical neurology teaching focuses on neuroscience subjects whereas the clinical years focus on clinical symptoms and signs related to neurological disease [7, 8]. The gap between pre‐clinical and clinical clerkship is a challenge [9] and some transition courses have been developed to bridge this gap [10].

Impactful change to a curriculum is challenging to demonstrate [11]. Medical education interventions lack uniformity in intervention design, methodology and standardized endpoints, making outcomes difficult to measure [12]. Previous studies have established that endpoints in neurology medical education are not well described and there is a paucity of randomized control trials [13]. Reporting guidelines have been developed to aid researchers in conducting educational interventions [14].

Exploring the methodology and design of neurology medical education is important prior to investigating problems or proposing solutions [15]. Educational design research (EDR) is a research approach used to address an issue when standard guidelines are unavailable [16, 17]. EDR describes “the process of understanding the theory behind empirical investigation in education, aiming to develop practical solutions to complex problems with the goal of informing the work of others” [17].

We performed a scoping literature review on undergraduate neurology education, with a focus on teaching content, research methodology, and outcome measures used. We describe the current knowledge/gaps in undergraduate neurology education literature. In so doing, we make recommendations for future studies and intervention design.

## 2 Methods

We performed a scoping review to map the literature on undergraduate neurology medical education. Scoping reviews are a knowledge synthesis used to map evidence and identify main concepts, theories, sources, and knowledge gaps on a specific topic [18]. We employed the methodology outlined by Arksey and O'Malley [19] with the amended recommendations by Levac et al. [20] and Joanna Briggs Institute [21].

The review protocol is registered on the Open Science Framework (OSF) and can be found at (https://osf.io/n7gck/). The scoping review is reported in accordance with Preferred Reporting Items for Systematic reviews and Meta‐Analyses extension for scoping reviews (PRISMA‐ScR) guidelines [18].

### 2.1 Step 1: Identifying the Research Question

The research questions for this review were developed in collaboration with the research team, and were outlined as follows:

1. What undergraduate neurology teaching methods are described in the literature?
2. How are undergraduate neurology educational interventions designed and delivered?
3. How are undergraduate neurology educational interventions assessed?

### 2.2 Step 2: Search Strategy: Identifying Relevant Studies

In collaboration with the university‐affiliated librarian, we drafted the following Boolean search query for our database search: ‘medical education’/exp. AND ‘undergraduate’ AND ‘neurology.’ The keywords included neurology, medical undergraduate education, medical students, and medical curriculum.

Legend: Adj = adjacent within × words in any order; Exp / = Medical subject heading; .mp. = title, abstract, subject field; ti, ab. = title/abstract field; Asterisk = Truncation.

The electronic databases of MEDLINE (Ovid), PubMed, Embase, and Education Resources Information Center (ERIC), were searched. Included article bibliographies were searched. Gray literature inclusive of government reports, institutional policy statements and conference proceedings were searched.

The literature search was conducted from database inception to July 2022. Only articles published in the English language were included. Participants included were undergraduate medical students of any age, gender, and geographical location. Studies which exclusively included qualified medical professionals and allied healthcare professionals were excluded. Only primary research was considered for this review.

### 2.3 Step 3: Study Selection

The combined database search was imported to ENDNOTE citation software [22]. Two reviewers (L.M. and E.M.) independently applied a screening tool to all retrieved article titles and abstracts to determine their eligibility for full article review (Appendix S1). Duplicates were excluded. A third‐party independent reviewer (S.B.) was consulted for any titles that remained under contention. For the selected titles and abstracts, full articles were distributed equally among four teams of two reviewers per team (L.M., E.M., J.M., A.E., C.H., H.L., K.B., and A.A.). Next, each reviewer read a designated number of articles for full review. Strict inclusion/exclusion criteria were applied to determine eligibility. For inclusion in the data extraction sheet, an article needs the following:

1. Focus on undergraduate neurology education curricula or interventions.
2. Focus on outcome measures used to assess the efficacy of undergraduate neurology educational curricula and interventions.
3. Focus on undergraduate medical students and not exclusively on residents or fellows.

We excluded books, book reviews, systematic, and scoping reviews as well as non‐English articles.

### 2.4 Step 4: Data Collection: Charting the Data

We employed Arksey and O′Malley's “descriptive analysis” [19] approach to data extraction, summarizing information from the selected articles on data extraction sheets. To validate this form, a pilot test was conducted after the revision and charting of 20 articles.

Eight reviewers (divided into four teams of two) (L.M., E.M., J.M., A.E., C.H., H.L., K.B., and A.A.) extracted the data. Each team of two cross‐checked one another's data extraction forms. Any conflicts were reviewed by a third party. The data extraction forms included demographic data (year of publication, location of publication, and participant characteristics), methodological categories (study methodology, intervention description, outcome measures, education format, and teaching methods).

Teaching methods were charted and definitions used in this review include the following [8]:

Didactic teaching: Traditional sense of learning practice involving an instructor‐centered classroom delivering large amounts of information with minimal student engagement.

Team‐based learning (TBL): Student‐centered learning with small groups of students having the opportunity to apply educational concepts through group activities with feedback from the instructor.

Case‐based learning (CBL): Teaching‐learning practice where clinical cases are employed to aid in traditional lectures.

Problem‐based learning (PBL): Modern learning combining complementary educational principles to form a clinical problem.

Simulation‐based learning (SBL): Man‐made illustration of a true world subject to achieve instructional motives via experiential learning.

Practical skills teaching: A broad term to summarize basic skills including: procedural, technical, physical examination, and clinical skills [23].

Patient‐based: Patient‐centered teaching including ward‐based and outpatient setting teaching.

Virtual teaching: e‐Learning methods involving online or electronic method of distribution and teaching.

Blending learning: Online and in‐person teaching combined.

Kirkpatrick level of evidence [24] was charted. The Kirkpatrick Model is an internationally recognized method of evaluating education programs. Kirkpatrick levels are Level 1 (reaction), Level 2A (knowledge assessment), Level 2B (performance assessment), Level 3 (behavior), Level 4A (change in system), and Level 4B (change in participant) [24]. If not formally cited in a study, reviewers used the Kirkpatrick Model to evaluate themes during the data extraction process.

### 2.5 Step 5: Collecting, Summarizing, and Reporting Results

Using the data extraction form, analysis of the selected studies are presented using descriptive methods (quantitative and qualitative). Data analysis involved quantitative frequency analysis and qualitative thematic analysis. Thematic analysis was performed via a theoretical semantic approach. This generated four main concepts to define the boundaries of outcome measures within this review (see results). The Kirkpatrick Model for Training Evaluation criteria was included as a data charting measure to (1) structure and organize outcome measure themes [24] and (2) evaluate intervention results. Study characteristics were collected including (type of study and year of publication), population characteristics and neurology education program characteristics (curriculum or study intervention, curriculum/intervention description, e.g., mode of delivery, educator, curriculum content, and teaching methodology).

Data collection methods and study outcome measures are documented from neurology education interventions to provide both a descriptive and numerical summary to answer our three research questions. Studies were classified according to the Oxford Centre for Evidence‐Based Medicine 2011 guidelines [25]. Results are reported below in descriptive‐narrative and tabular formats.

## 3 Results

### 3.1 Descriptive Results

#### 3.1.1 Included and Excluded Studies

**FIGURE 1:**
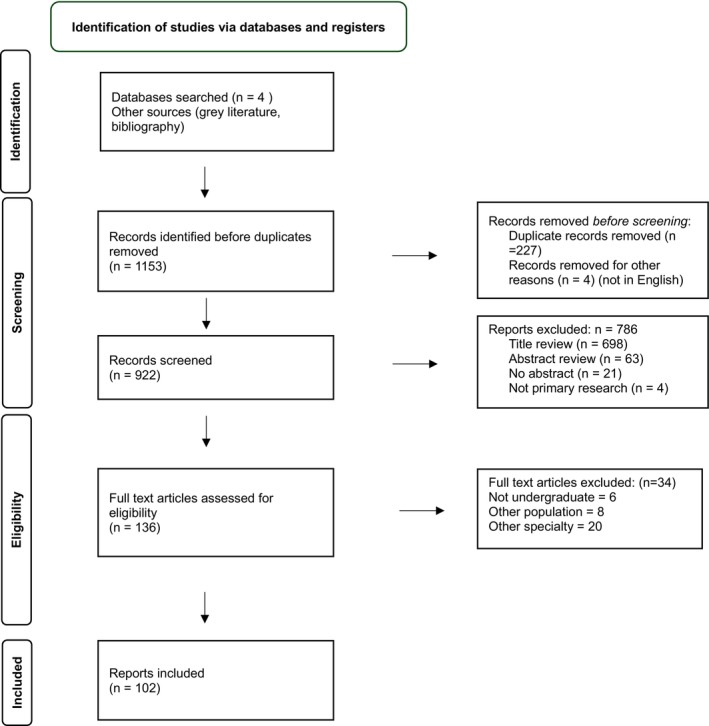
Preferred Reporting Items for Systematic Review and Meta‐analyses (PRISMA)—Scoping Review flow chart.

The process of study selection and literature search are presented in Figure 1. From 1153 identified articles, 136 articles were identified for full‐text review following initial exclusions. A further 34 articles were excluded following full‐text review; this was mainly because the studies did not include undergraduate medical students or exclusively targeted another medical specialty. A total of 102 articles were identified for data extraction and included in the data analysis.

#### 3.1.2 Article Characteristics

Of the 102 included studies, a majority of studies (79%, *n* = 82/102) were published since 2005 (See Table 1) [26, 27, 28, 29, 30, 31, 32, 33, 34, 35, 36, 37, 38, 39, 40, 41, 42, 43, 44, 45, 46, 47, 48, 49, 50, 51, 52, 53, 54, 55, 56, 57, 58, 59, 60, 61, 62, 63, 64, 65, 66, 67, 68, 69, 70, 71, 72, 73, 74, 75, 76, 77, 78, 79, 80, 81, 82, 83, 84, 85, 86, 87, 88, 89, 90, 91, 92, 93, 94, 95, 96, 97, 98, 99, 100, 101, 102, 103, 104, 105, 106, 107]. Most papers were from the United States of America (46% *n* = 47/102). All articles included were primary research. Articles were classified according to the Oxford Evidence‐Based Medicine (OCEBM) level of evidence [25] (See Table 1). No articles where level one evidence as systematic reviews were not included in this scoping review. Eleven per cent (*n* = 11/102) of studies reached level two evidence (randomized controlled trial, 10% (*n* = 10/102) achieved Level 3 (case series and historically controlled cohort) and 56% (*n* = 57/102) achieved Level 4 (mechanism‐based‐reasoning), and 23% (*n* = 24/102) achieved Level 5 evidence). A narrative description of all neurology education intervention and non‐interventional studies, including teaching methods, study outcomes, and results, are presented in Table S1.

**TABLE 1:** All article characteristics (*n* = 102).

	*N* (%)
*Location*	
All articles	102 (100)
United States of America^a^	47 (46)
United Kingdom^a^	9 (9%)
Germany	7 (7%)
Singapore	5 (5%)
Canada	5 (5%)
Australia^a^	4 (4%)
France	3 (4%)
Brazil	2 (2%)
Ireland^a^	2 (1%)
Colombia	1 (1%)
West Indies	2 (2%)
Saudi Arabia	1 (1%)
Finland	1 (1%)
Greece	1 (1%)
Turkey	1 (1%)
Iran	1 (1%)
The Netherlands	1 (1%)
Denmark	1 (1%)
Nigeria	1 (1%)
Poland	1 (1%)
Hong Kong	1 (1%)
Korea	1 (1%)
Multi‐national studies	4 (4%)
*Year*	
2020–present	26 (25%)
2016–2019	24 (24%)
2010–2015	23 (22%)
2005–2009	9 (9%)
2000–2004	10 (10%)
1977–1999	10 (10%)
*Article type (level of evidence)*	
Randomized trial (2)	11 (11%)
Non‐randomized controlled cohort (3)	10 (10%)
Case series, case–control (4)	57 (56%)
Mechanism based reasoning (5)	24 (23%)

^a^ Denotes articles participating in multi‐national studies.

#### 3.1.3 What Undergraduate Neurology Teaching Methods Are Described in the Literature?

Sixty‐one articles described neurology education interventional studies [26, 27, 28, 30, 31, 33, 36, 37, 38, 40, 42, 43, 44, 46, 47, 48, 50, 53, 54, 56, 58, 60, 63, 64, 65, 66, 67, 76, 77, 78, 82, 83, 84, 85, 90, 91, 92, 93, 94, 95, 96, 97, 98, 99, 104, 105, 106, 108, 109, 110, 111, 112, 113, 114, 115, 116, 117, 118, 119] and 41 articles were non‐interventional [29, 32, 34, 35, 39, 49, 51, 55, 57, 62, 68, 69, 72, 73, 74, 75, 79, 80, 81, 86, 87, 88, 100, 101, 103, 107, 120, 121, 122, 123, 124, 125, 126, 127, 128, 129, 130, 131, 132, 133, 134].

**FIGURE 2:**
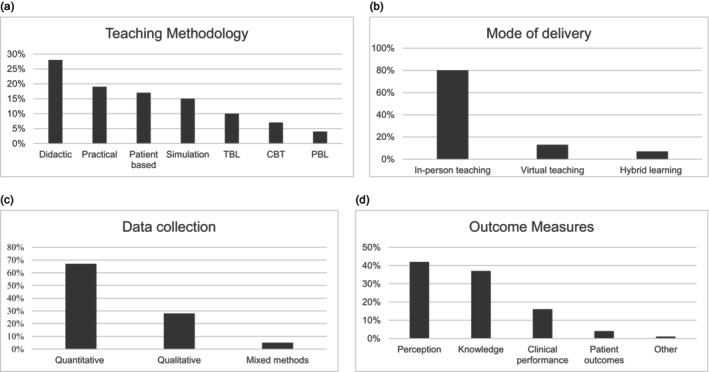
(a) Teaching methodology quantitative frequency analysis in interventional studies (*n* = 61). Didactic teaching methods (28%) either alone or in combination with other methods within interventions were the most utilized. (b) Teaching mode of delivery quantitative frequency analysis in interventional studies (*n* = 61). Eighty percent of studies utilized in‐person teaching in interventions. (c) Data collection methodology evaluated via quantitative frequency analysis in interventional studies (*n* = 61). The majority of studies utilized quantitative analysis (67%). (d) Quantitative frequency analysis was performed of the outcome measures identified via thematic analysis. The majority of studies utilized student perception (42%) and student knowledge (37%).

In intervention studies, both single‐method and multi‐modal methods (≥ 2 teaching methods) were used across studies (summarized Table 2.1 and illustrated in Figure 2a). The relative frequency of teaching methodologies was calculated (Table 2.2). Overall, didactic teaching had the highest overall frequency in interventional studies. In studies where two teaching methods were used, the most common combination was didactic and simulation (*n* = 7/37) [27, 44, 58, 71, 96, 105, 106, 112].

**TABLE 2.1:** Teaching methods in interventional studies.

Teaching method	Percentage	*N*	References
Multi‐modal^a^	61%	37	[24, 27, 28, 31, 42, 44, 47, 48, 49, 53, 55, 56, 58, 60, 64, 66, 68, 71, 78, 84, 85, 89, 90, 91, 93, 94, 95, 97, 105, 106, 107, 114, 119, 120, 121, 122, 135]
Single‐method	39%	24	[26, 33, 38, 40, 41, 43, 50, 54, 65, 67, 69, 76, 86, 92, 96, 99, 104, 113, 115, 116, 117, 118, 126]

^a^ Multi‐modal methods: refer to studies using two or more teaching methods.

**TABLE 2.2:** Teaching method frequencies in interventional studies.

Teaching method	Percentage	*N*	References
Didactic	28%	32	[27, 31, 33, 38, 40, 44, 47, 48, 51, 53, 56, 58, 64, 66, 67, 68, 71, 78, 84, 85, 91, 93, 95, 96, 97, 105, 106, 107, 119, 120, 121, 126]
Practical	19%	21	[31, 42, 47, 54, 56, 66, 68, 78, 83, 89, 90, 93, 94, 95, 105, 113, 115, 116, 118, 120, 122]
Patient‐based	17%	18	[31, 42, 43, 48, 55, 58, 66, 89, 90, 92, 94, 95, 97, 99, 114, 117, 119, 120, 135]
Simulation	15%	17	[27, 31, 44, 50, 55, 60, 65, 71, 76, 85, 91, 93, 105, 106, 107, 114, 121, 122]
Team‐based learning (TBL)	10%	11	[31, 41, 49, 53, 55, 64, 78, 84, 86, 87, 93]
Case‐based learning (CBL)	7%	8	[24, 26, 28, 31, 48, 49, 53, 104]
Problem‐based learning (PBL)	4%	5	[24, 28, 48, 53, 60]

*Note:* The percentages represent the frequency of each teaching method used across all interventional studies.

Non‐interventional studies categories are detailed in Table 3. Of the 41 non‐intervention studies, a majority (34%, *n* = 14/41) described an institutional curriculum, 20% (*n* = 8/41) described models of teaching, 17% (*n* = 7/41) reported validation of formative assessments, 17% (*n* = 7/41) described student or educator opinions, and 10% (*n* = 4/41) were published organizational reports. One study surveyed the level of knowledge in a neurology subtopic across multiple institutions.

**TABLE 3:** Categories of non‐intervention studies.

Category	Percentage	*N*	References
Institutional curriculum	34%	14	[29, 34, 39, 46, 62, 77, 82, 88, 110, 125, 131, 132, 134, 135]
Models of teaching	20%	8	[30, 35, 36, 63, 75, 111, 112, 129]
Validation of formative assessments	17%	7	[32, 37, 57, 74, 80, 100, 109]
Student or educator opinions	17%	7	[72, 79, 81, 101, 123, 124, 133]
Organizational reports	10%	4	[103, 108, 127, 130]
Knowledge survey across institutions	2%	1	[69]

*Note:* Percentages represent the frequency of each category in non‐intervention studies.

#### 3.1.4 How Are Undergraduate Neurology Educational Interventions Designed and Delivered?

Of the articles describing a neurology educational intervention, a majority (80%, *n* = 49/61) used in‐person teaching, 13% (*n* = 8/6) used a virtual mode of delivery, and 7% (*n* = 4/61) used hybrid modes of delivery (combining virtual and in‐person teaching). Findings are summarized in Table 4.1 and illustrated in Figure 2b.

**TABLE 4.1:** Modes of delivery for neurology educational interventions (*N* = 61).

Mode of delivery	*n* (%)	References
In‐person teaching	49 (80%)	[24, 31, 38, 40, 41, 42, 43, 44, 47, 48, 49, 50, 51, 53, 54, 55, 56, 58, 60, 64, 65, 66, 67, 68, 71, 78, 84, 86, 89, 90, 92, 94, 95, 96, 97, 99, 105, 107, 113, 114, 115, 116, 117, 118, 120, 121, 122, 126, 135]
Virtual mode	8 (13%)	[26, 27, 28, 76, 85, 87, 104, 119]
Hybrid (virtual and in‐person)	4 (7%)	[33, 91, 93, 106]

Of the studies that used either virtual or hybrid modes of delivery, 36% (*n* = 4/11) of studies took place before 2019, and 64% (*n* = 7/11) took place during the period of the COVID‐19 pandemic (2019–2022) (see Table 4.2).

**TABLE 4.2:** Timeline of virtual and hybrid neurology educational interventions (*N* = 11).

Period	*n* (%)	References
Before 2019	4 (36%)	[26, 27, 28, 33, 91]
During COVID‐19 pandemic (2019–2022)	7 (64%)	[85, 87, 93, 104, 106, 119]

Most interventions used classroom‐based teaching models (54%, *n* = 33/61), followed by clinical clerkships (16%, *n* = 10/61), and virtual e‐modules (16%, *n* = 10/61). A minority of studies employed workshops (7%, *n* = 4/61), a dedicated neurology teaching day (3%, *n* = 2/61) or the addition of a neurology committee or task force (2%, *n* = 1/61). One interventional study utilized both classroom‐based and virtual teaching (see Table 4.3).

**TABLE 4.3:** Educational settings in neurology educational interventions (*N* = 61).

Setting	*n* (%)	References
Classroom‐based	33 (54%)	[24, 28, 31, 38, 40, 41, 43, 44, 47, 48, 49, 54, 55, 58, 64, 65, 66, 67, 78, 84, 86, 91, 96, 99, 105, 107, 113, 114, 115, 118, 121, 122, 126]
Clinical clerkships	10 (16%)	[42, 71, 89, 90, 92, 94, 95, 117, 120, 135]
Virtual e‐modules	10 (16%)	[26, 27, 51, 76, 85, 87, 93, 104, 106, 119]
Workshops	4 (7%)	[50, 53, 60, 68]
Dedicated neurology day	2 (3%)	[97, 116]
Neurology committee/task force	1 (2%)	[56]
Classroom and virtual	1 (2%)	[33]

*Note:* See Figure 2b for a visual breakdown of delivery modes and settings.

The curriculum content was categorized by subject, subtopic and subspeciality. The relative frequency of curriculum content among interventional studies was analyzed. General neurology (46%) was the most frequently documented curricular content, followed by clinical reasoning (31%), neurology subtopics (14%), and subspecialties (9%).

Subtopics and subspecialities included neuroanatomy, neurology practical skills (e.g., lumbar puncture), peripheral nervous system, movement disorders, epilepsy, neurocritical care, neurovascular, neuropathology, neurophysiology, neurorehabilitation, and neurosurgery.

The educators delivering the intervention were documented. A neurologist was formally identified within the article text as the primary educator in 34% (*n* = 21/61) [33, 42, 43, 47, 56, 58, 70, 71, 77, 82, 90, 92, 94, 95, 96, 98, 99, 109, 112, 117, 119] of interventions. Other educators included clinical tutors, lecturers, academic professors in medicine and neurosurgery, peer‐led teaching, and resident‐led teaching.

#### 3.1.5 How Are Undergraduate Neurology Educational Interventions Assessed*?*

Of the interventional studies, 67% (*n* = 41/61) utilized quantitative data collection measures, 28% (*n* = 17/61) used mixed methods, and a minority (5%, *n* = 3/61) used qualitative methods only. Twenty‐three percent (*n* = 14/61) used a validated scale and 39% (*n* = 24/61) used institutional formative assessments. Self‐reported surveys were the most used modality for student perception evaluation (49%, *n* = 30/61), the majority of which (83%, *n* = 25/30) used a Likert scale. Four studies used focus groups as a data collection measure. See Tables 5.1, 5.2, 5.3, 5.4 and Figure 2c.

**TABLE 5.1:** Assessment methods of undergraduate neurology educational interventions (*N* = 61).

Assessment method	*n* (%)	References
Quantitative data collection	41 (67%)	[28, 31, 38, 40, 41, 42, 43, 47, 48, 49, 50, 51, 53, 54, 56, 60, 64, 65, 66, 67, 78, 85, 86, 87, 90, 91, 92, 93, 95, 96, 99, 106, 107, 114, 117, 118, 119, 121, 122, 126, 135]
Mixed methods	17 (28%)	[24, 27, 33, 44, 55, 58, 68, 71, 76, 84, 94, 97, 104, 105, 113, 115, 116]
Qualitative methods only	3 (5%)	[26, 70, 120]

*Note:* See Figure 2c for a visual representation of assessment methods.

**TABLE 5.2:** Target population in neurology educational interventions (*N* = 61).

Target population	*n* (%)	References
Medical students only	42 (69%)	[27, 28, 33, 38, 41, 43, 44, 47, 48, 49, 50, 51, 56, 60, 64, 66, 67, 68, 71, 76, 78, 84, 86, 87, 89, 90, 91, 93, 94, 95, 96, 97, 99, 104, 107, 113, 115, 116, 117, 118, 119, 120, 122, 126]
Medical students and educators	13 (21%)	[24, 26, 31, 40, 42, 53, 85, 105, 106, 121, 136]
Medical students and patients	5 (8%)	[54, 58, 114, 135]
Students, educators, and patients	1 (2%)	[55]
Educators only	1 (2%)	[92]

**TABLE 5.3:** Data collection instruments in neurology educational interventions (*N* = 61).

Instrument	*n* (%)	References
Validated scale	14 (23%)	[41, 42, 64, 65, 66, 67, 68, 87, 90, 92, 114, 117, 122, 135]
Institutional formative assessments	24 (39%)	[24, 27, 28, 38, 44, 55, 65, 66, 78, 84, 86, 90, 92, 94, 95, 98, 99, 106, 107, 114, 117, 118, 126, 135]

**TABLE 5.4:** Student perception evaluation methods in neurology educational interventions (*N* = 61).

Evaluation method	*n* (%)	References
Self‐reported surveys	30 (49%)	[24, 27, 28, 31, 33, 38, 40, 41, 43, 44, 48, 49, 50, 51, 53, 54, 55, 58, 60, 64, 65, 66, 68, 71, 76, 78, 84, 85, 86, 87, 93, 94, 95, 96, 97, 104, 105, 106, 113, 114, 115, 116, 119, 120, 121, 122]
Surveys using Likert scale	25 (83% of surveys)	[31, 43, 44, 49, 51, 55, 60, 66, 68, 71, 85, 87, 93, 94, 96, 97, 106, 116, 121, 122]
Focus groups	4	[26, 58, 76, 89]

The target population in interventional articles was charted. Medical students alone were the target population in most intervention studies (69%, *n* = 42/61). Twenty‐one percent (*n* = 13/61) collected data from medical students and educators whereas 8% (*n* = 5/61) collected data from medical students and real or simulated patients. One interventional study, (2%, *n* = 1/61), collected data from students, educators, and patients [115]. One study collected data from educators alone (2%, *n* = 1/61) [114].

The four categories of outcome measures identified via thematic analysis are as follows: (1) student perception of teaching or subject matter (2) student knowledge of subject matter, (3) student clinical performance, and (4) patient‐based outcomes. The relative frequency of thematic outcome measures among interventional studies was analyzed. Student perception (42%) was the most frequently used thematic outcome measure, followed by student knowledge (37%), clinical performance (16%), patient outcomes (4%), and other outcomes (neurology residency application rate and attendance) (1%) (See Figure 2d). Only one study utilized all four outcome measures [31]. One study's outcomes did not fit into the four thematic subcategories. This study measured student engagement with the intervention by attendance and number of applications to neurology residency in the calendar year [91].

### 3.2 Kirkpatrick's Model of Training Evaluation [24]

Of the intervention studies, 3% (3%, *n* = 2/61) [67, 92] of articles formally evaluated the intervention using the Kirkpatrick framework [24]. The reviewers independently evaluated the four themes identified within outcome measures under the Kirkpatrick criteria level of evidence. Most studies achieved up to two levels (69%, *n* = 42/61), 23% (*n* = 14/61) achieved only one level, and 8% (*n* = 5/61) achieved three or more. Most educational interventions (70%, *n* = 43/61) employed Level 1 (reaction). Level 1 measured with Level 2B (21%, *n* = 13/61) was the most utilized combination.

## 4 Discussion

We sought to map the current literature on undergraduate neurology education to formulate recommendations for future neurology undergraduate education and research. Specifically, we focused on undergraduate neurology education teaching content, research methodology, and outcome measures used.

Neurology undergraduate education research has steadily increased since 2010 with over a quarter of included articles published within the last 2 years. Despite this increased interest, methodological guidelines are not clearly defined. This gap in the literature represents an opportunity to develop evidence‐based undergraduate neurology teaching programs.

Our review identified the following main results: (1) The main teaching styles currently in use are didactic and experiential. (2) Evidence‐based design of interventions is heterogenous and (3) the outcome measures most frequently used are student perception and knowledge.

Multimodal teaching made of up the majority of teaching methods in this review.

Didactic teaching includes the instructor‐focused and lecture‐based teaching format used traditionally in medical education. Experiential learning (EL) is “a process whereby knowledge is created through the transformation of experience” and consists of “concrete experience, reflective observation, abstract conceptualization and active experimentation” [135]. EL has been linked with improved motivation by allowing learners to interact and critically evaluate course material as it applies to the real world [136, 137]. Our findings align with the move toward experiential learning within medical institutions [136, 138]. Medical school curricula are guided by institutional and national training bodies. The European Academy of Neurology (EAN) [139] and American Academy of Neurology (AAN) [7, 140] include clerkship and course director resource recommendations at both undergraduate and post graduate levels. Guidelines are referenced from national and international standards based on majority expert consensus through postgraduate training bodies such as the European Union of Medical Specialists (EUMS) and European Training Requirements for Neurology (EAN).

The heterogeneity in the structure and design of undergraduate neurology teaching interventions is consistent with previous reviews [141, 142]. We also found variation in the study outcomes defined in the articles. Synthesis and interpretation of results is impacted when research design varies and creates difficulty in the validation and replication of interventions.

Randomized control trials (RCTs) are considered the gold standard of research design through a structure which limits bias or systematic errors [143, 144]. In this review, a minority of included studies utilized a randomized control design (Table 1). The lack of RCT design used in neurology education research likely reflects the constraints of adhering to medical school curricular requirements. Medical schools offer equal education opportunities to all students. This limits the feasibility of creating a control group. To address this issue, research designs such as a randomized cross‐over trial have been used [144] where all participants receive two interventions sequentially over two periods, the order of which is randomized [145, 146]. In medical education, this study design allows participants to act as their own control and thus maintains equality in educational opportunities [144].

In‐person and classroom based teaching were the most common modes of delivery in educational interventions in our review. Of the studies which used virtual and hybrid teaching, a majority were published during the SARS‐CoVID‐19 pandemic. This likely reflects the impact of mandatory social distancing on the education environment. Hybrid educational techniques reported better academic outcomes compared with online‐only education in this timeframe [147, 148]. Virtual‐only teaching techniques has its limitations, including limited technical resources, varied supervision, and student compliance [149]. Future research could compare the hybrid model of teaching versus established in‐person‐only curriculum.

The role of neurologists and neurology trainees in neurology medical education is well supported [140, 150], although only a minority of articles cited a qualified neurologist as the primary educator. This likely represents under‐reporting as some papers may have omitted identifying the primary educator's qualifications within the text. Neurologists as role‐models can shape future professional development [85]. Resident‐led teaching has also been shown to improve overall student experience within neurology clerkships and is a pillar in the American Academy of Neurology (AAN) core curriculum guidelines [85, 140, 150, 151].

Neurologists are under‐resourced in both clinical and academic settings [152], and the ratio of students to educational staff is increasing [153, 154, 155]. Educational interventions that facilitate education to a large cohort of students through practical and experiential teaching modalities should be encouraged.

Standardized program evaluation techniques are an evolving process in the medical education research design community [156]. The Best Evidence Medical Education collaboration (BEME Guide) has cited Kirkpatrick levels as one potential target for evaluating medical education interventions [156]. However, achieving all levels of Kirkpatrick framework can be a challenge for educators [141]. Indeed, only a minority of studies formally evaluated undergraduate neurology teaching interventions using the Kirkpatrick criteria [24].

Level 4A (change in system) and 4B (change in participant) require longitudinal data of patient outcomes and post‐graduate clinical performance. This poses a significant resource burden and may not always be practical. However, understanding the impact of undergraduate teachings on future clinical practice is valuable information that may influence and optimize innovative undergraduate medical education.

A student's learning experience is considered equally important as knowledge acquisition when targeting learning objective goals [157, 158]. In our review, student knowledge and perception evaluations were the main themes used across study outcome measures (Figure 2d). Evaluation of outcomes using the Kirkpatrick Level of Evidence criteria [24] also revealed a predominance among interventions for learner reactions (Kirkpatrick Level 1) and assessments of knowledge and performance (Kirkpatrick Level 2A and Level 2B). The psychological theory of Dewey advocates that an educational system should distribute equal weight to both the subject matter and the interests and experiences of the student [159]. Knowledge assessment can help identify student comprehension of the subject matter and direct student learning and inform learning goals [158, 160]. Although, student evaluation of teaching (SET) is also a valuable quality assessment tool in higher medical education engagement. SET can inform a program's effectiveness [161] and has been shown to have positive correlation with student learning [161, 162, 163].

### 4.1 Recommendations for Undergraduate Neurology Education and Research Design

We have structured our recommendations for neurology undergraduate education and research design using a PICO framework [164, 165] (See Figure S1). Author recommendations are based on (1) majority consensus of results informed by through the Delphi method [166, 167, 168, 169], and (2) comparison of the most up to date guidelines on educational research design recommendations [11, 17, 170].

Population: Medical students as the primary target population. Medical students are a relevant, actionable, and appropriate cohort for education research design. The majority of studies utilize medical students to assess the efficacy of neurology education interventions at the highest level of evidence criterion.

Intervention: Content should focus on general neurology core principles and updates to an original curriculum. Teaching via a hybrid education program is supported. Teaching methodology should include combinations of multimodal, didactic, and experiential learning techniques designed and delivered by educators trained in neurology.

Comparison: A randomized cross‐over design using mixed method data collection of primary and secondary endpoints including all four outcome measure themes outlined below.

Outcomes: Outcome themes should include student knowledge, clinical performance, perception, and patient outcomes. Other outcome measures to consider are change in institution (system) in order to fulfill all Kirkpatrick levels [24].

### 4.2 Limitations

This review is limited to four databases and as such we may not have identified all potential studies for inclusion. To capture all relevant studies, the gray literature, and bibliographies of included articles were reviewed. Only neurology undergraduate education studies were included. Studies exclusively examining other neuroscience subspecialties were not included for review. This likely limits the breadth of interventional studies relevant to neuroscience. As the data were heterogenous the authors used qualitative thematic analysis for categorizing outcome measures which were then evaluated using the Kirkpatrick criteria [24]. This may have impacted data accuracy reporting due to human error and inherent bias. This was mitigated to an extent through data re‐checking by a second‐ and third‐author party prior to analyses.

## 5 Conclusion

Neurology medical education intervention studies have increased in the last 10 years. Undergraduate neurology education studies are limited by lack of standardized outcome measures. Most education interventions are taught in person via didactic methods combined with experiential learning techniques (simulation, patient, and practical teaching). Virtual and hybrid teaching were more common during the Sars‐COV‐19 pandemic. The most common outcome measure themes identified are knowledge and perception. The level of Kirkpatrick framework evaluated was congruent with the themes identified among outcome measures. The Kirkpatrick model of education is under‐utilized in neurology undergraduate education interventions. Standardized guidelines for neurology educational interventions are needed to allow researchers to implement evidence‐based change into undergraduate neurology curricula.

## Author Contributions

**L. McElligott:** conceptualization, investigation, funding acquisition, writing – original draft, methodology, validation, writing – review and editing, software, formal analysis, resources, data curation. **A. Ardilouze:** writing – review and editing, data curation. **J. Moloney:** data curation. **A. ElSheikhId:** data curation. **C. Healy:** data curation. **H. Leahy:** data curation. **K. Babatunde:** data curation. **C. Cahir:** formal analysis, software, validation. **P. Murphy:** data curation, software, methodology. **N. Delanty:** funding acquisition, writing – review and editing. **N. McElvaney:** writing – review and editing. **S. Byrne:** writing – review and editing. **E. McGovern:** supervision, writing – review and editing, conceptualization, project administration.

## Disclosure

Digital artwork created by primary author and publication license obtained via BioRender.

## Ethics Statement

The authors have nothing to report.

## Conflicts of Interest

The authors declare no conflicts of interest.

## Previous Presentations

This review was previously presented as a poster at the Association of British Neurologists (ABN) and Irish Neuroscience Association (INA) Joint Meeting in Belfast, Northern Ireland on Friday May 12, 2023.

## Supporting information


Table S1.

Appendix S1.

Figure S1.

## Data Availability

The authors have nothing to report.
