# Supplementary material for: Neurology Undergraduate Medical Education: A Scoping Review
Source: Eur J Neurol. 2025 Mar 13;32(3):e70061. doi: 10.1111/ene.70061 (PMC11904807; doi:10.1111/ene.70061)
Supplement: Supplementary file 1 — Table S1. [file ENE-32-e70061-s003.docx]

Table S1. Summary description of all studies (n = 102)

|  | **Article title** | **Author** | **Study design** | **Country** | **Year** | **Summary** |
| --- | --- | --- | --- | --- | --- | --- |
| **1** | Assessment of neurological clinical management reasoning in medical students | Lukas | Retrospective cohort study | USA | 2014 | Neurology clerkship described. Student knowledge and performance in diagnostic and therapeutic management versus supportive management assessed. Observed structured clinical examination (OSCE) and USA National Board of Medical Examiners (NBME) used. Student knowledge of diagnostic and acute therapy significantly higher than supportive. Therapeutic and supportive management scores significantly correlate with NBME score. |
| **2** | Neuroanatomy learning: augmented reality vs. cross sections | Henssen | Randomized control trial | The Netherlands | 2019 | Virtual anatomy learning resource described. Resource compared to traditional cross-sectional anatomy. Student knowledge assessed. Knowledge scores significantly higher in cross-sectional anatomy group versus virtual anatomy. |
| **3** | Development and Assessment of a New 3D Neuroanatomy Teaching Tool for MRI Training | Drapkin | Randomized control blinded trial | USA | 2013 | Virtual three-dimensional (3D) neuroanatomy radiology teaching in third year medical students described. 3D teaching compared to standard two-dimensional teaching to assess efficacy Student knowledge of magnetic resonance imaging (MRI) structure identification assessed in both groups. Knowledge improved significantly for neuroanatomical internal structures in 3D group. Most students would recommend 3D teaching to other students. |
| **4** | Effect of feedback from standardized patients on medical students’ performance and perceptions of the neurological examination | Park | Randomized control trial unblinded | Korea, USA | 2012 | Standardized patient (SP) feedback as an assessment for final year medical students described. SP feedback compared to educator feedback. Student clinical performance of neurological examination assessed before and after in both groups’ Feedback combined with educator feedback resulted in significantly higher scores than educator alone. |
| **5** | Teaching pediatric epilepsy to medical students: A randomized  crossover trial | Bye | Randomized cross over trial | Australia | 2009 | Interactive lecture and computerized tutorials for pediatric epilepsy for third- and fourth-year medical students described. Student clinical performance and perception assessed before and after teaching. Both teaching methods found to be effective with no significant difference in clinical performance in both groups. Interactive lecture perceived significantly more effective for teaching on clinical performance. No significant differences were found between interest or willingness to learn between both groups |
| **6** | A Brazilian original pedagogical approach to the teaching of neurology | Magalhães | Randomized cross over trial | Brazil | 2014 | Workshop on clinical decisions and diagnoses for stroke education described. Study objective examined workshop versus traditional didactic curriculum. Sample size of 68 medical students in 4^th^ year. Student knowledge and perception assessed. Educator perception of program also assessed. Student knowledge significantly improved in workshop group. Majority of student and educators felt workshop was beneficial. Major drawback included the long duration of time for students to complete workshop. |
| **7** | Evaluating a Bedside Tool for Neuroanatomical Localization  with Extended-Matching Questions | Tan | Randomized control study | Singapore | 2017 | Neuroanatomical localization tool (NLT) for bedside teaching and assessment method using extended matching questions (EMQ) for neurolocalisation for fourth year medical students is described. Student knowledge assessed via EMQ before and after in both groups. NLT resulted in significantly higher knowledge scores on EMQ. Validation of EMQ in assessing neurolocalisation skills. |
| **8** | Comparing computer-assisted learning activities for learning clinical neuroscience: a randomized control trial | Rajan | Randomized control trial | UK | 2022 | E-module using virtual clinical cases and graphics described. E-module compared to Wikipedia. Student knowledge and perception assessed in both groups. E-module rated significantly more engaging, useful, and enjoyable (factors identified: interactivity, clinical cases). No significant improvement in knowledge in either group. |
| **9** | A randomized trial of hypothesis-driven vs screening neurologic examination | Kamal | Randomized control trial | USA | 2011 | Hypothesis driven neurological examination teaching described in fourth year medical students. Hypothesis driven examination compared to screening neurologic examination teaching to assess efficacy and accuracy of neurological examination technique. Student performance and feedback assessed. Student confidence in their performance significantly improved in both groups. There was a significant improvement in sensitivity but not specificity of performance in hypothesis driven examination. There was a significant improvement in specificity with the traditional screening approach. |
| **10** | A Novel Three-Dimensional Tool for Teaching Human Neuroanatomy | Estevez | Randomized control trial | USA | 2010 | Practical modelling for anatomy teaching using 3D model described. Study objective evaluated if a physical modelling exercise improved students’ 3D understanding of the brain and compared it to 2D modelling. Student knowledge and perception assessed. Significant change in student knowledge for 3D modelling the majority preferred 3D model for learning. |
| **11** | Randomized Comparison Between Traditional and Traditional Plus Interactive Web-Based Methods for Teaching Seizure Disorders | Ochoa | Randomized control trial | USA | 2008 | Neurology clerkship using learning resources of e-learning interactive tutorial for epilepsy teaching described. Study objectives to determine the benefits of incorporating Web-based interactive methodology into a neurology clerkship to enhance the teaching about seizure disorders to 3rd-year medical students. Clerkship compared to standard syllabus (textbook). Student knowledge and feedback assessed. Student knowledge significantly better in e-learning group. No significant difference in satisfaction ratings of either resource. |
| **12** | Teaching neurology to medical students with a simplified version of team-based learning | Brich | Randomized cross over trial | Germany | 2017 | Team based learning (TBL) versus small group seminar teaching for clinical knowledge and reasoning. Sample size of 122 third- and fourth-year medical students. Student knowledge assessed using key feature problem (KFPE) solving and multiple-choice questions (MCQ). Student perception of teaching assessed via questionnaire No significant differences found in MCQ results. Student KFPE for topic of acute altered mental status significantly better after TBL versus small group seminars. Students preferred TBL overall. |
| **13** | Does improved problem-based teaching influence students’ knowledge at the end of their neurology elective? An observational study of 40 students | Heckmann | Non randomized control cohort study | Germany | 2003 | Problem based learning (PBL) for clinical, data interpretation and practical skills in neurology described. Study objective to evaluate the gain in theoretical and practical skills of sixth year students trained by PBL from the results of sixth year students trained by the conventional standard teaching method. Student knowledge and perception assessed. PBL group showed significant improvement in knowledge compared to standard method. Both groups rated high satisfaction with teaching |
| **14** | Effectiveness of non-bedside teaching during the COVID_19 pandemic: a quasi-experimental study | Heitmann | Non randomized control quasi-experimental study | Germany | 2022 | Virtual teaching during COVID-19 described. Study objective to compare effectiveness of non-bedside teaching versus clinical bedside teaching. Student and educator feedback assessed. Students rated the clinical in-person teaching as significantly better and self-rating of knowledge improved in both groups. Educators rated that the student knowledge acquired in virtual teaching was significantly better. |
| **15** | A controlled study of team-based learning for undergraduate clinical neurology education | Tan | Randomized cross over trial | Singapore | 2011 | TBL for the neurological examination and clinical localization in third year medical students described. TBL group compared to passive learning group. Student knowledge and feedback assessed. Knowledge was significantly better in TBL versus passive learning. Students reported more engagement in TBL group. The effect of TBL was found to be larger in academically weaker students. |
| **16** | An observational study of an approach to accommodate a fourth year to third year neurology clerkship curricular transition | Kraakevik | Nonrandomized control | USA | 2020 | Competency based ‘opt out’ approach for a mandatory 4th year neurology clerkship described.  Students who did not attend clerkship were compared to students who attended. Student knowledge assessed before and after in both groups. Student knowledge did not differ between groups. The majority (58%) opted out of the neurological clerkship. |
| **17** | The impact of formal instruction in clinical examination skills on medical student performance - the example of peripheral nervous system examination | Fox | Nonrandomized control | UK | 2001 | Practical teaching of peripheral nervous system examination described during a rheumatology elective. Practical teaching compared to standard teaching. Study objective to evaluate if formal instruction in PNS exam translates to improved OSCE (observed structured clinical examination) scores. Student performance assessed. OSCE scores significantly improved with practical teaching of peripheral nervous system examination. |
| **18** | Usefulness of videotape instruction in an academic department of neurology | Kaufman | Nonrandomized control | USA | 1983 | Videos of clinical patients as learning resource to identify neurological conditions described. Video teaching compared to traditional curriculum in fourth year medical students Student knowledge assessed. Significant improvement in group who used videos in identifying conditions versus traditional teaching. |
| **19** | Attitudes and Performance of Third- vs Fourth-Year Neurology Clerkship Students | Dewey | Nonrandomized single blind controlled trial/ cross sectional | USA | 2010 | Neurology clerkship in third and fourth year described. Student knowledge, performance and perception assessed between two groups. No significant difference in knowledge or performance between the groups Enthusiasm for neurological learning significantly higher in third year students. |
| **20** | Experiential Learning: Transforming Theory into Practice through the Parkinson’s Disease Buddy Program | Cumberland | Quasi-experimental cohort study | USA | 2019 | Experiential service-learning initiative and learning objectives for Parkinson’s disease described. Student knowledge and student attitudes assessed before and after. Experiential learning showed significant improvement in student knowledge scores and attitudes in relation to Parkinson’s Disease. The majority of students indicated they would recommend the program and participate again. |
| **21** | Ultrasound's Impact on Preclinical Medical Student Neurology Unit Grades | Shah | Quasi-experimental non randomized control study | USA | 2020 | Practical teaching using an ultrasound (US) workshop for first- and second-year medical students described. Study objective to determine if academic performance in final examinations differed between optional US workshop versus no workshop. Student knowledge assessed. There was no significant difference in knowledge scores in either group |
| **22** | Comparison of Team-Based Learning versus Traditional Lectures in Neuroanatomy: Medical Student Knowledge and Satisfaction | Rezende | Quasi-experimental study - post-test with nonrandomized control | Brazil | 2019 | Team-based learning in neuroanatomy described. TBL compared to traditional lectures. Student knowledge acquisition and perception assessed. TBL was significantly associated with greater perception of acceptance, motivation, and methodology was perceived to be able to integrate clinical and basic sciences. There was no significant change in knowledge between groups. |
| **23** | Education research: A qualitative study on Student Perceptions of Neurology and Psychiatry Clerkship Integration | Mowchun | Case series, Prospective qualitative | USA | 2021 | Neurology and psychiatry integrated clerkship described. Student perceptions of the feasibility of integration assessed by theme. Three major themes arose: (1) combining the clerkships was not favorable as students need sufficient time to delve deeper into each discipline; (2) students did not observe an integrated clinical approach by faculty; (3) there is positive value to making links between neurology and psychiatry for effective patient care. |
| **24** | Linking neuroscience theory to practice helping overcome student fear of neurology | Hudson | Case series | Australia | 2006 | Case based in-person teaching (CBT) described. Student clinical performance, feedback and educator feedback assessed to assess efficacy of CBT. No significant change found in clinical performance. Feedback themes of CBT (theory-practice link, smaller size class, practical) identified as most helpful. Themes of barriers identified: difficulty teaching in CBL format, large volume of content and language barrier. CBT was subsequently included in the standard curriculum. |
| **25** | Neurological examination course in an interactive webinar as a solution during a pandemic. An overview of the implementation, optimization as well as critical considerations | Oster | Case series | Germany | 2021 | Virtual teaching described. Virtual teaching program, based on bedside teaching, developed during the pandemic to convert bedside teaching to an online format. Student satisfaction assessed. Overall students satisfied with teaching. |
| **26** | Evaluation of an Interactive Case-based Online Network (ICON) in a Problem Based Learning Environment | Nathoo | Case study | USA | 2005 | Interactive case-based online network (ICON) during problem-based learning curriculum described. Student perception and utilization of ICON with educator feedback assessed. ICON was used sufficiently by both faculty and students. Students identified themes of real-time engagement, stronger relationships with faculty, increased accountability to the tutorial group and self-selected pace as the most beneficial characteristics of the ICON. Faculty identified enhanced collaboration with students. Issues identified with ICON by both groups included: time management and reliance on good faculty mentorship. |
| **27** | Expanding medical student interaction in neurology with a re-designed student interest group in neurology (sign) chapter | Gummi | Cohort study historically controlled | USA | 2021 | Neurology committee for undergraduate students described (SIGN chapter). Student interest and engagement with SIGN and residency applications assessed. Data compared to previous year. Significant increase in student enrolment, engagement, and interest in neurology career in SIGN group. No significant increase in neurology residency applications found. |
| **28** | Implementing Change in Neuroanatomy Education: Organization, Evolution, and Assessment of a Near-Peer Teaching Program in an Undergraduate Medical School in Greece | Karamaroudis | Cross sectional analysis | Greece | 2020 | Near-Peer led (PT) teaching for second to sixth year medical students in neuroanatomy course described. Student knowledge and perception of peer led teaching assessed. Lower student knowledge scores were significantly associated with lower rating of PT teaching. Significant increase in acknowledgement of contribution of peers across all years. |
| **29** | Standardized patient outcomes trial (SPOT) in neurology | Safdieh | Cross sectional study with historical control | USA | 2011 | Practical teaching using standardized patient (SP) session on neurological examination described. Study objectives to determine whether using SPs to teach the neurological examination affects neurological examination skills in the long term. SP teaching compared to didactic teaching in previous years. Student performance and perception assessed. Student performance significantly improved in SP group versus previous years of didactic teaching. Students reported SP as valuable contribution to their learning. |
| **30** | Miming neurological syndromes improves medical student's long-term retention and delayed recall of neurology | Roze | Historical control study | France | 2018 | An innovative simulation-based neurological semiology teaching program described. Simulation semiology teaching versus standard curriculum alone. Student knowledge assessed. Significant improvement in short term and long-term retention of knowledge in simulation teaching group. |
| **31** | Improving the neurological exam skills of medical students | Moore | Historically controlled | Canada | 2012 | Clinical neurological examination consisting of additional time in practicing skills described. Teaching compared to standard amount of time. Student performance assessed after additional teaching. Performance significantly better in additional time teaching group. |
| **32** | Medical students’ experience of emotions and success in neurological studies – What do they tell us? | Ansakorpi | Multi-center case series/ cross sectional | Finland | 2017 | Four-week clinical hospital course described. Student perception assessed before and after. Barriers to learning identified before: lack of knowledge, inability to link clinical exam to basic neuroscience, anxiety, and lack of interest. Reasons for barriers: subject complexity, and difficulty linking neuroscience to clinical exam findings. The most significant factors to reduce barriers: small group teaching, real patient exposure, educator expertise, improving self-confidence. |
| **33** | The effect of face-to-face teaching on student knowledge and satisfaction in an undergraduate neuroanatomy course | Whillier | Nonrandomized control is historical | Australia | 2013 | Didactic teaching with additional teaching hours described. Teaching compared to standard time format for neuroanatomy. Student performance and perception assessed. No significant difference found in knowledge. Self-rating of knowledge was significantly higher in additional teaching group. Students rated additional teaching significantly higher than standard amount. |
| **34** | Tackling student neurophobia in neurosciences block with team-based learning | Anwar | Nonrandomized historical control follows up study | Saudi Arabia | 2015 | TBL neurology curriculum described. TBL compared to previous academic curriculum. Student knowledge and feedback assessed. TBL significantly improved student knowledge in male students more than female and low-average students compared to top students. Students preferred TBL overall. |
| **35** | Where’s the logic in neurologic education? | Gelb | Observational - retrospective case series | USA | 2003 | Digital learning resource for clinical hospital-based teaching described. Digital resource compared to print version. Student knowledge assessed in both groups. No significant difference in knowledge between digital and print resource. |
| **36** | Making the neurology clerkship more effective:  can e-Textbook facilitate learning? | Jao | Observational case series | USA | 2013 | Hospital based neurology clinical elective utilizing a digital neurology resource in place of printed material is described. Student satisfaction assessed over a time period. Student satisfaction rates rose over time. When utilizing an abbreviated timeline for elective and digital learning resource. |
| **37** | Implementation of Integrated Learning Program in neurosciences during first year of traditional medical course: Perception of students and faculty | Ghosh | Observational case series | India | 2008 | Integrated learning program(ILP = case-based learning, seminar, patient exposure) for neurology education described. ILP compared to traditional curriculum. Student satisfaction and educator feedback on ILP assessed. The majority of students rated the program good to excellent. Majority of students did not find that ILP would help them at professional examinations. Majority of faculty agreed that program improved understanding of basic sciences. Both groups found ILP to be satisfactory overall. |
| **38** | Is Virtual Team-Based Learning Feasible and Effective in Teaching Neurolocalisation? | Ong | Observational cohort study | Singapore | 2021 | Virtual TBL module described. Study objectives to assess final year medical students’ knowledge and reactions to virtual TBL via Kirkpatrick criteria. No significant improvement in knowledge assessment found. The majority of students were satisfied with the virtual TBL module. |
| **39** | Medical Students' Viewpoint Regarding the Integrated Module of Basal  Ganglia | Mehr | Observational cross sectional | Iran | 2011 | Elective workshop of case-based and multi-disciplinary team-based learning for basal ganglia described Student knowledge and satisfaction assessed before and after. Significant improvement in both knowledge and student satisfaction post workshop. |
| **40** | ‘The Move’, an innovative simulation-based medical education program using roleplay to teach neurological semiology: Students’ and teachers’ perceptions | Roze | Observational post test case study | France | 2016 | Role-play simulation training program ‘The Move’ using neurology semiology described. The Move is compared to standard curriculum. Student neurophobia (fear of neurology, confidence in neurological examination, motivation to learn and perception of learning) assessed. Majority of students demonstrated improvements in components of Neurophobia post-teaching. Overall satisfaction with The Move was high. |
| **41** | Teaching bioelectricity and neurophysiology to medical students using Lab AXON simulations | Monteiro | Observational, quasi-experimental | UK | 2021 | Simulation teaching using Lab AXON (bioelectricity and neurophysiology) education described. Student knowledge and perception assessed. High overall knowledge score post Lab AXON teaching. Majority of students agreed it consolidated their knowledge. Most difficult topics included sodium gated channel activation and refractory period. Most manageable topic was all or non-principle of action potential. |
| **42** | The effectiveness of TBL with real patients in neurology education in terms of knowledge retention, in-class engagement, and learner reactions | Alimoglu | Prospective historical control | Turkey | 2016 | TBL utilizing real neurological patients described. TBL compared to lecture-based teaching in neurology clerkship. Student knowledge via formative assessment, knowledge retention, engagement and satisfaction assessed. Patient feedback on TBL was also assessed. There was no significant difference in knowledge scores in formative assessment in either group. There was significant improvement in knowledge retention in TBL group only. Average satisfaction was overall higher in TBL group. Patients were highly satisfied with TBL participation. |
| **43** | Active learning in neuroscience: a manipulative to simulate visual field defects | Li | Quasi experimental | USA | 2016 | Problem based learning for first year medical students in visual field neurological examination described. Student knowledge and perception of confidence assessed. Significant improvement in perceived confidence in visual field examination and knowledge of visual field deficits. |
| **44** | An evaluation of optimal tutorial methodologies for neurology teaching at undergraduate level | Togher | Quasi experimental cohort study historically controlled | Ireland | 2021 | Short intensive course (1 week) of neurology education in final year medical student cohort described. Short course compared to series weekly tutorials. Student confidence in neurological knowledge assessed before and after. No significant difference in ranking of confidence in neurology knowledge and clinical performance in either group. |
| **45** | The role of residents in medical students’ neurology education: current status and future perspectives | Keser | Quasi experimental cross-sectional study | USA | 2020 | Resident led teaching program for third year medical students described. Student perception of teaching and effectiveness of resident teaching assessed before and after. Confidence in management and interest in neurology residency significantly increased after teaching Significant correlation between student perception of rotation experience and resident teaching effectiveness. |
| **46** | Teaching Undergraduate Medical Students, The Neurological Exam | Laguna | Quasi experimental cross-sectional study | USA | 1978 | Neurology clerkship consisting of dedicated teaching of the neurological examination on a normal individual. Student clinical performance and perception assessed before and after. Clinical performance significantly improved post teaching. Length of time in program also noted to improved performance scores. Majority of students found the dedicated normal exam teaching extremely useful. |
| **47** | A Required, Combined Neurology-Physical Medicine and Rehabilitation Clerkship Addresses Clinical and Health Systems Knowledge Gaps for Fourth-Year Medical Students | Curtis | Quasi-experimental case series | USA | 2021 | Neurology clinical elective combined with neurology rehabilitation rotation described. Student performance and confidence assessed before and after. Significant improvement in clinical performance post elective. Students felt more confident in examination skills, the teaching created a positive learning environment, and the combination of programs increased patient exposure. |
| **48** | Broadening learning communities during COVID-19: developing a curricular framework for telemedicine education in neurology | Gummer son | Quasi-experimental case series | USA | 2021 | Telemedicine education elective framework for neurological clinical presentations (Virtual Patient Rounds in Neurology) described. Student and faculty confidence with telemedicine assessed before and after. Students reported a significant improvement in history taking and performing a telemedicine neurological exam post elective. Faculty reported increased confidence with teaching clinical medicine virtually. |
| **49** | An educational initiative to improve medical student awareness about brain death | Lewis | Quasi-experimental case series | USA | 2018 | Multimodal teaching of didactic and simulation teaching on clinical skills for pronouncing brain death described. Student knowledge and confidence assessed before and after. Knowledge and confidence in pronouncing brain death significantly improved post teaching. |
| **50** | Novel Dissection of the Central Nervous System to Bridge Gross Anatomy and Neuroscience for an Integrated Medical Curriculum | Hlavac | Quasi-experimental case series | USA | 2017 | Two teaching interventions piloted in two separate student groups described. The first is a laboratory skills session using wet pathology dissection of central nervous system then piloted in graduate students. The second is teaching using preserved plasticated specimens teaching neurolocalization for undergraduates on clinical elective. Student feedback assessed before and after. Majority of undergraduate students recommended that CNS prosection be introduced prior to clinical rotations. Majority of students in both groups found pathology teaching useful and recommended it for further students. |
| **51** | Objective-structured teaching of undergraduate neurology | Scherokman | Quasi-experimental case series | USA | 1985 | Clinical neurological inpatient hospital teaching described. Inpatient teaching compared to neurology outpatients with both groups receiving the same learning objective list prior Student knowledge and student feedback assessed before and after. Significant improvement in knowledge post teaching in both groups. No significant difference in knowledge between in-patient and outpatient teaching. Students found learning objective list useful. |
| **52** | Training in Neurology: Neuro Day | Frey | Quasi-experimental cross-sectional study | USA | 2021 | One-day workshop for neurology described for first year medical students. Study objectives to determine whether increased patient interaction, exposure to the neurologic examination, and access to positive neurology mentors increase interest in neurology. Student perception and neurophobia assessed. Significant increase in interest in neurology, self-reported level of knowledge, and comfort with neurology post workshop. Significant decrease in fear of neurology post workshop. All students would recommend to peers. |
| **53** | Applied Neuroanatomy Elective to Reinforce and Promote Engagement with Neurosensory Pathways Using Interactive and Artistic Activities | Dao | Quasi-experimental cross-sectional study | USA | 2014 | Study objective to assess if an elective course to reinforce and integrate neurosensory pathways is an effective complement to a standard neuroscience course. A “near-peer” led teaching program that used physical modalities and clinical vignettes to better understand neurological pathways for 1^st^ year medical students described. Student perception of teaching assessed. Familiarity and comfort with neurological disease improved after peer-led teaching significantly. |
| **54** | Medical student attitudes and educational interventions to prevent neurophobia: a longitudinal study. | Shiels | Quasi-experimental longitudinal study | Grenada West Indies | 2017 | TBL, CBT and PBL for neurological education in first year medical students described. Student knowledge, perception and neurophobia assessed at start of year and repeated in 2^nd^ year. Significant improvement in neuroscience knowledge and confidence level in second year but also a significant increase in level of neurophobia in second year. No significant change in perception of neurology or desire to pursue in a career. |
| **55** | A simulated hospital: an effective teaching tool during the COVID-19 pandemic | Toro | Quasi-experimental study with historical control | Colombia | 2022 | Virtual simulation of hospital in neurology clerkship for fifth year medical students described. Compared to standard in-person curriculum. Student knowledge and perception assessed. No significant change in knowledge between groups. Students found the simulated hospital facilitated their learning and allowed for greater interaction with educator. Barriers identified included concentration levels via virtual modality were more difficult to maintain. |
| **56** | Neuroscience Curriculum Changes and Outcomes | Holden | Quasi-experimental study with historical control | USA | 2012 | Neurology clinical elective with new curriculum learning objectives described. Student knowledge assessed and compared to national scores before and after. No significant improvement in knowledge post elective. Results using new curriculum comparable to historic national scores. |
| **57** | NeuroQ: A neurophobia screening tool assesses how roleplay challenges neurophobia | McGovern | Quasi-experimental two-part monocentric prospective cross-sectional study | France | 2021 | Novel simulation role-playing teaching program for neurology semiology ‘The Move’ described. Compared to traditional curriculum. Student neurophobia assessed via validated scale before and after. Significant improvement in neurophobia level post The Move. |
| **58** | Outcomes From Building System Course ware for Teaching and Testing in a Discipline-Based Human Structure Curriculum | Reilly | Retrospective | USA | 2011 | Pre-clinical teaching in first year students of neuroanatomy using didactic system-based and interactive lectures described. Compared to traditional anatomy teaching over the course of 3 years Student knowledge and perception of learning objectives assessed retrospectively. Significant improvement in knowledge for both groups. Students found benefit over all with learning objectives in interactive teaching |
| **59** | Undergraduate neurology teaching: Comparison of an inpatient versus outpatient clinical setting | Martins | Retrospective case control | Portugal | 2021 | Clinical neurology in-patient teaching described. Compared to neurological outpatient only teaching. Student knowledge and performance assessed in both groups post teaching. Both knowledge and performance significantly higher in inpatient teaching versus outpatient only. Inpatient setting was an independent predictor of both knowledge and clinical performance. |
| **60** | Different Formats for a Neurology Clerkship Do Not Influence Written Examination Scores | Gunderson | Retrospective case control | USA | 2003 | Neurology and other sub-specialty in-hospital rotations described over a 2-year period. Compared to non-neurological specialty clerkships over same time period. Student knowledge assessed. No significant difference in knowledge in either group |
| **61** | Undergraduate neuroscience education | Resnick | Letter to Editor/ descriptive | USA | 2000 | Six-week neurology curriculum with additional neurosurgical teaching week described. Longer curriculum compared to traditional (3- to 4-week) neurology rotation. Student performance assessed. Students did not perform better in standard shorter curriculum. |
| **62** | The neurology and neurosurgery interest group (NANSIG) - 10 years of cultivating interest in clinical neurosciences | Park | Descriptive | Ireland, UK | 2022 | Organization report on current curriculum, organizational structure and current research projects being undertaken in neurology by the Neurology and Neurosurgery Interest Group (NANSIG) |
| **63** | Multiple choice versus open ended questions in advanced clinical neuroanatomy: using a national neuroanatomy assessment to investigate variability in performance using different question types | Merzoughui | Cross sectional | UK | 2021 | Formative assessment evaluation of open ended versus single best answer (SBA) for clinical neuroanatomy described. Student knowledge assessed. Significantly higher average knowledge score in SBA group for all topics except the cerebellum. |
| **64** | Introduction of the Modified Neuroanatomy Motivation Questionnaire and Its Role in Comparing Medical Student Attitudes Towards Learning Neuroanatomy Between Neuro-enthusiasts and Standard Students | Hall | Cohort study | UK | 2021 | Elective neuroscience competition for students participating in neurology extracurricular courses described. Compared to students not in additional courses. Student learning motivation assessed in both groups Students participating in extra course showed differences in motivation to learn neuroanatomy. Themes identified for differences included: increased career motivation, personal relevance, intrinsic motivation, and assessment anxiety. |
| **65** | Training in Neurology: How Lessons Learned on Teaching, Well-being, and Telemedicine During the COVID-19 Pandemic Can Shape the Future of Neurology Education | Sandrone | Report from authorities - cross sectional analysis | USA | 2021 | Report on challenges and lessons learned in neurology education since COVID 19 and the necessary changes and adaptations to virtual learning and telemedicine.  Learning suggests that hybrid education can improve neurology undergraduate education. |
| **66** | The current state of headache medicine education in the United States and Canada: An observational, survey-based study of neurology clerkship directors and curriculum deans | Pace | Observational study - cross sectional | USA and Canada | 2021 | Undergraduate curriculum for headache medicine described Neurologist opinion on curriculum needs and issues assessed. Identified issues: access to clerkships, time constraints, volume overload in curriculum, resource restrictions, lack of student interest. Overall findings conclude that headache medicine is taught pre-clinically, and headache education has improved from an educator perspective. |
| **67** | A Diverse Specialty: What Students Teach Us About Neurology and “Neurophobia” | Moore | Prospective qualitative study | Canada | 2020 | Clinical elective in neurology described for fourth year medical students. Study objectives examined questions including what they learn, experience and what would they change within a clinical elective. Student perception assessed. Pre and post elective the highly rated theme were “localization”. Post elective the students rated the importance of physical exam higher. Students surprised about the scope of practice and expressed interest in acute neurology (hospital based) setting exposure. |
| **68** | Neurological teaching in times of crisis | Biesalski | Observation case study | Germany | 2020 | Virtual e-learning resource for clinical neurology described. Compared to in person teaching. Educator feedback and confidence with digital teaching assessed. Majority of educators rated clinical in-person teaching better. Reported high level of self-reported confidence with digital modalities but majority described increased burden of workload and time expenditure issues. |
| **69** | From bedside to website: A neurological clinical teaching experience | Tsang | Opinion from authority | Hong Kong | 2020 | Virtual learning resource using live patients described. Student feedback assessed. Students found observing live patients via virtual modality useful. |
| **70** | A novel longitudinal framework aimed at improving the teaching of the neurologic examination | Bornkamm | Cross sectional | Germany | 2019 | Framework for neurological examination teaching described. Survey of neurologists on the essential components of neurological examination assessed. Recommendations included a shorter component list for novice learners compared to advanced learners |
| **71** | Validation and perception of a key feature problem examination in neurology. | Grumer | Observational case study | Germany | 2019 | Formative assessment using focused neurology semiology linked to neurological examination described. Student perception of formative assessment examined. Overall new assessment was validated by previous formative assessments and was perceived well by students. |
| **72** | Attitudes towards neurology among medical undergraduates | Pokrysko-Dragan | Qualitative case study | Poland | 2019 | Clinical neurology curriculum for final year medical students described. Student perception assessed. Majority perceived neurology to be interesting and important to medical education and ranked it highly as a potential future career. Barriers identified included neurology subject difficulty. |
| **73** | Preventing neurophobia: remodeling neurology education for 21^st^ century medical students through effective pedagogical strategies for “neurophilia” | Shelley | Descriptive study | India, Australia | 2018 | Traditional knowledge-based curriculum adaptations to a student-centered outcome based and competency driven education program described. Author assessment of curriculum and recommendations outlined. Identified issues included: lack of criteria in curriculum, neuroanatomy taught with lack of clinical correlation, lack of access to patients for students, lack of formal neuro-mentorship program, and lack of objective assessments. Recommendations included: using Miller’s pyramid to remodel effective neurological pedagogical strategies and improve competence in bedside clinical teaching. |
| **74** | Education Research: Difficult conversations in neurology: Lessons learned from medical students | Lemmon | Cross sectional concurrent mixed methods | USA | 2018 | Clinical neurology clerkship with a focus on difficult clinical case conversations described. Student perception assessed. Students identified pre-conversation planning as well as a debriefing session invaluable but infrequently done. Most students desired bedside training in communication skills. Barriers identified included: student uncertainty and fear during difficult conversations. |
| **75** | Entrustable professional activities - A useful concept for neurology education | Horak | Report from authorities | USA | 2018 | Describes Entrustable Professional Activities (EPA) as an assessment tool for clinical clerkships. Tool used to give student feedback after each clinical learning episode. EPA tool to provide formal mechanisms to assess student level of perception required. |
| **76** | Incorporating sleep medicine content into medical school through neuroscience core curricula | Salas | Report from authorities | USA | 2018 | Curriculum proposing sleep medicine teaching introduction into the neuroscience curriculum. The curriculum proposed 2–4 hours per year of didactic teaching, flipped-classroom, clinical teaching, and online teaching-modalities. |
| **77** | Knowledge of medical students about epilepsy: Need for a change | Adikaibe | Cross sectional observational | Nigeria | 2017 | Neurology clinical elective described. Compared to no elective. Knowledge outcome assessed. Students that participated in elective, those who had witnessed seizures and the older demographics of students showed significant improvements in knowledge scores relating to epilepsy. |
| **78** | Medical Undergraduate Survey on Headache Education in Singapore: Knowledge, Perceptions, and Assessment of Unmet Needs | Ong | Descriptive observational study | Singapore | 2017 | Curriculum and syllabus for headache medicine compared to standard diabetes curriculum. Student perception and exposure to learning assessed. headache medicine had significantly less exposure compared to diabetes in terms of average hours overall. Majority of students expressed exposure to learning was inadequate for headache medicine. |
| **79** | Breadth versus volume: Neurology outpatient clinic cases in medical education | Albert | Descriptive study | USA | 2016 | Neurology hospital based clinical cases were correlated to formative assessments. Student knowledge and clinical performance assessed. Clinical exposure in hospital was correlated significantly with case examples in formative assessments. |
| **80** | Neurology Clerkship goals and their effect on learning and satisfaction | Strowd | Prospective cohort study | USA | 2016 | Neurology compulsory clerkship for consecutive second to fourth year medical students described. Student knowledge and feedback on goal setting assessed. Students who reported achieving their goals demonstrated significantly higher adjusted NBME shelf examination scores, clerkship satisfaction, and observed self-directed learning behaviors. |
| **81** | Neurology objective structured clinical examination reliability using generalizability theory | Blood | Observational study | USA | 2015 | Validation of neurology objective structured clinical examination (OSCE) formative assessment. Results demonstrated that students should participate in three cases to increase reliability of scores. |
| **82** | A competency-based longitudinal core curriculum in medical neuroscience | Merlin | Report from authorities | USA | 2014 | Competency-based longitudinal core curriculum in medical neuroscience described. Curriculum included 1) case logs of clinical experiences 2) assesses tools for learning objectives and achievable goals 3) observed examination and knowledge assessment using national scores 4) simulation and patient exposure, 5) practice-based learning 6) systems-based structure 7) professionalism assessments using formative knowledge and clinical observation. |
| **83** | Incorporating Simulation Technology Into a Neurology Clerkship | Ermak | Descriptive | USA | 2013 | Simulation training for clinical neurology described. Educator opinion on case exposures of students assessed. Simulation training exposes more clinical cases to students. |
| **84** | Introducing neurological examination for medical undergraduates—how I do it | Wiles | Descriptive | UK | 2012 | Description of in-person neurological examination tutorials from educator. Advises for consensus on recommended exam components. |
| **85** | Assessment of Genetics Knowledge and Skills in Medical Students: Insight for a Clinical Neurogenetics Curriculum | Pearl | Observational/ feasibility study | USA | 2011 | Neurology clerkship in fourth year medical students described. Student knowledge and self-assessment assessed on level of understanding of basic and clinical genetics concepts, clinical skills in genetics, and preferred learning styles. Results used to inform curriculum. Students self-reported competence with basic science from pre-clinical education was better than clinical competence from later years. Knowledge of inheritance patterns was better than genetic counselling knowledge. Awareness of practicalities in neurogenetics (websites, referrals) was low amongst most students. |
| **86** | Guidelines for the organization of headache education in Europe: the headache school II | Jensen | Report from committee | Denmark | 2010 | Guidelines, a day-to-day program, and a multiple-choice test battery outlined for headache medicine to meet international standards, CME, and secure patronage. |
| **87** | The essential neurologic examination: What should medical students be taught? | Moore | Observational study | Canada | 2009 | Opinion survey evaluating the differences in what neurologists consider essential elements to the neurological examination compared to medical students. Found majority consensus amongst neurologists and medical students matched |
| **88** | Improving undergraduate clinical neurology bedside teaching: opening the magic circle | Emsley | Descriptive study | UK | 2009 | Experiential learning theory (ELT) for neurology bedside teaching described. Opinion of educator assessed on student engagement. Educator found student engagement improved using ELT. |
| **89** | Evaluation of the assessment and grading of medical students on a neurology clerkship | Schmahmann | Prospective cross sectional | USA | 2008 | Neurology clerkship using a formative assessment tool using clinical bed-side examination described. Compared to previous validated measures of assessment. Student knowledge and perception assessed. Bedside examination helped limit grade inflation but did not predict final grade determined. Useful as a complementary assessment tool. |
| **90** | Using videotaped vignettes to teach medical students to perform the neurologic examination | Erle | Letter to Editor | Singapore | 2006 | Multi-media didactic neurological examination focused lectures with clinical video vignettes followed by practical session described. Virtual teaching successfully incorporated into curriculum. |
| **91** | Strategies to Attract Medical Students to the Specialty of Child Neurology | Werner | Descriptive study | USA | 2004 | Pre-clinical and clinic neuroscience curriculum described. Pediatric neurologists from medical schools that graduate the most pediatric residents assessed. Factors that contribute to more pediatric resident’s graduation: earlier introduction of neuroscience curriculum in first and second year, higher academic reputation in pediatrics and neurology as well as overall reputation. |
| **92** | Viewpoint: Evidence-Guided Education: Patients’ Outcome Data Should Influence Our Teaching Priorities | Glick | Opinion from authority | USA | 2003 | Clinical data interpretation and outcome measures using patient outcomes described. Educator opinion assessed. Concludes patient outcomes should be used for evidence-based education. |
| **93** | Introduction of Patient Video Clips into Computer-Based Testing: Effects on Item Statistics and Reliability Estimates | Lieberman | Cohort study | USA | 2003 | Digital formative assessment tool with patient videos compared to text-based assessment described. Digital patient video assessment. Validated and reliable as formative assessment compared to standard. |
| **94** | The neurology clerkship core curriculum | Gelb | Report from committee | USA | 2002 | Core curriculum for neurology undergraduate education with focus on neurological examination components described. Focus on symptom based versus disease-based learning. Recommend teaching skills to be applied relevant to the setting of acute versus chronic presentation. |
| **95** | Medical neurobiology: do we teach neurobiology in a format that is relevant to the clinical setting? | Haines | Descriptive study | USA | 2002 | Neuroanatomy teaching program utilizing radiological correlates to link to clinical setting described. |
| **96** | How much Neurology Should a Medical Student Learn? A Position Statement of the AAN Undergraduate Education Subcommittee | Charles | Report from committee | USA | 1999 | Education model to integrate neuroscience and clinical neurology. Four components described: 1) Integrated first year neuroscience course focusing on the normal structure and function of the nervous system. 2) Integrated second year course focusing on the diseases of the nervous system and its pathophysiology. 3) Integrated third year neurology clerkship, at least four weeks in length. 4) Fourth year elective time for a neurology sub-internship, subspecialty rotation, or ambulatory care experience. |
| **97** | Statement on medical education in neurology | Menken | Report from committee | UK | 1994 | Description of an education model to integrate neuroscience and clinical neurology: 1) Integrated first year neuroscience course focusing on the normal structure and function of the nervous system. 2) Integrated second year course focusing on the diseases of the nervous system and its pathophysiology. 3) Integrated third year neurology clerkship, at least four weeks in length. 4) Fourth year elective time for a neurology sub-internship, subspecialty rotation, or ambulatory care experience. |
| **98** | Epilepsy education in medical schools: Report of the American Epilepsy Society Committee on Medical Student Education | Devinsky | Report from committee | USA | 1993 | Committee suggestions for neurology undergraduate curriculum: basic knowledge of neuroanatomy and neurophysiology is fundamental to understanding neurological disease. Difficult to form a uniform approach to teaching; basic guidelines can help achieve minimum level of training. |
| **99** | Medical education and the challenge of neurological disability | Ward | Descriptive study | UK | 1992 | Description that suggests three topics for neurology medical undergraduate education as follows: 1) clinical competence, 2) communication skills, 3) and professional values.  Advises medical education can contribute to improving services for people with neurological disability and could design curriculum on the whole patient care experience in addition to knowledge. |
| **100** | A survey of undergraduate teaching of clinical neurology in the United Kingdom 1990 | Wilkinson | Report from committee | UK | 1991 | Survey of students undergoing didactic and clinical hospital-based teaching. Students found didactic teaching is overly represented compared to clinical. The ratio of neurologists to students was reported as inadequate. |
| **101** | Comparing Students’ Feedback about Clinical Instruction with their performances | Anderson | Cross sectional observational | USA | 1991 | Neurology clinical hospital-based teaching compared across four sites described. Student clinical performance and feedback assessed. Statistically significant difference in one site o in clinical performance. |
| **102** | Relevance in Undergraduate Neurological Teaching | Murray | Cross sectional | Canada | 1977 | Novel curriculum on general neurology. General practitioners (GPs) perception assessed to see what commonly presented to GP. Inpatient neurological presentations recorded and compared to neuroscience curriculum. A ‘bad attitude’ towards neurology was identified in GPs. Outlined curriculum suggestions based on common inpatient and GP clinical presentations |
