# Supplementary material for: Neurology Undergraduate Medical Education: A Scoping Review
Source: Eur J Neurol. 2025 Mar 13;32(3):e70061. doi: 10.1111/ene.70061 (PMC11904807; doi:10.1111/ene.70061)
Supplement: Supplementary file 2 — Appendix S1. [file ENE-32-e70061-s002.docx]

# [
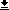
](https://www.editorialmanager.com/acadmed/download.aspx?id=375491&guid=cdf05445-cd10-4d92-ab90-da253ae9c051&scheme=1)Supplemental Digital Appendix 1

## All included studies citations (n =102).

1. Laguna JF, Stillman PL. Teaching undergraduate medical students. The neurological examination. Journal of Medical Education. 1978;53(12):990-2.
2. Kaufman DM, Kaufman RG. Usefulness of Videotape Instruction in an Academic Department of Neurology. 1983;58(6):474-78.
3. Scherokman BJ, Gunderson CH. Objective-structured teaching of undergraduate neurology.

Medical Education. 1985;19(6):452-5.

1. Anderson DC, Harris IB, Allen S, Satran L, Bland CJ, Davis-Feickert JA, et al. Comparing students' feedback about clinical instruction with their performances. Acad Med. 1991;66(1):29-34.
2. Wilkinson IM. A survey of undergraduate teaching of clinical neurology in the United Kingdom 1990. J Neurol Neurosurg Psychiatry. 1991;54(3):266-8.
3. Ward CD. Medical education and the challenge of neurological disability. J Neurol Neurosurg Psychiatry. 1992;55 Suppl:54-8.
4. Devinsky O, Lowenstein D, Bromfield E, Duchowny M, Smith DB. Epilepsy education in medical schools: report of the American Epilepsy Society Committee on Medical Student Education. Epilepsia. 1993;34(5):809-11.
5. Menken M, Hopkins A, Walton H. Statement on medical education in neurology. Working Group for Neurology and the World Federation for Medical Education. Med Educ. 1994;28(4):271-4.
6. Murray TJ. Relevance in undergraduate neurological teaching. 1997.
7. Charles PD, Scherokman B, Jozefowicz RF. How much neurology should a medical student learn? a position statement of the AAN Undergraduate Education Subcommittee. Academic medicine : journal of the Association of American Medical Colleges. 1999;74(1):23-6.
8. Resnick DK, Ramirez LF. Neuroscience education of undergraduate medical students. Part II: Outcome improvement. Journal of Neurosurgery. 2000;92(4):642-5.
9. Fox R, Dacre J, McLure C. The impact of formal instruction in clinical examination skills on medical student performance -- the example of peripheral nervous system examination. Med Educ. 2001;35(4):371-3.
10. Gelb DJ, Gunderson CH, Henry KA, Kirshner HS, Jozefowicz RF, Consortium of Neurology Clerkship D, et al. The neurology clerkship core curriculum. Neurology. 2002;58(6):849-52.
11. Haines DE, Hutchins JB, Lynch JC. Medical neurobiology: do we teach neurobiology in a format that is relevant to the clinical setting? Anat Rec. 2002;269(2):99-106.
12. Gelb DJ. Where's the logic in neurologic education? Experimental neurology. 2003;184 Suppl 1:S48-52.
13. Gunderson CH, Dougherty DS, Ford GC, Schwab K. Different formats for a neurology clerkship do not influence written examination scores. 2003.
14. Lieberman SA, Frye AW, Litwins SD, Rasmusson KA, Boulet JR. Introduction of patient video clips into computer-based testing: effects on item statistics and reliability estimates. Acad Med. 2003;78(10 Suppl):S48-51.
15. Werner RM, Polsky D. Strategies to attract medical students to the specialty of child neurology.

Pediatric neurology. 2004;30(1):35-8.

1. Glick TH. Evidence-guided education: patients' outcome data should influence our teaching priorities. Academic medicine : journal of the Association of American Medical Colleges. 2005;80(2):147-51.
2. Jao CS, Brint SU, Hier DB. Making the neurology clerkship more effective: can e-Textbook facilitate learning? Neurol Res. 2005;27(7):762-7.
3. Nathoo AN, Goldhoff P, Quattrochi JJ. Evaluation of an Interactive Case-Based Online Network (ICON) in a Problem Based Learning Environment. 2005;10(3):215-30.
4. Hudson JN. Linking neuroscience theory to practice to help overcome student fear of neurology.

Med Teach. 2006;28(7):651-3.

1. Lim EC, Ong BK, Seet RC. Using videotaped vignettes to teach medical students to perform the neurologic examination. J Gen Intern Med. 2006;21(1):101.
2. Ghosh S, Pandya HV. Implementation of Integrated Learning Program in neurosciences during first year of traditional medical course: Perception of students and faculty. BMC Medical Education. 2008;8(1):44.
3. Heckmann JG, Dutsch M, Rauch C, Lang C, Weih M, Schwab S. Effects of peer-assisted training during the neurology clerkship: a randomized controlled study. Eur J Neurol. 2008;15(12):1365- 70.
4. Ochoa JG, Wludyka P. Randomized comparison between traditional and traditional plus interactive Web-based methods for teaching seizure disorders. Teaching and learning in medicine. 2008;20(2):114-7.
5. Schmahmann JD, Neal M, MacMore J. Evaluation of the assessment and grading of medical students on a neurology clerkship. Neurology. 2008;70(9):706-12.
6. Bye AM, Connolly AM, Farrar M, Lawson JA, Lonergan A. Teaching paediatric epilepsy to medical students: A randomised crossover trial. J Paediatr Child Health. 2009;45(12):727-30.
7. Emsley H. Improving undergraduate clinical neurology bedside teaching: opening the magic circle. The Clinical Teacher. 2009;6(3):172-6.
8. Moore FG, Chalk C. The essential neurologic examination: what should medical students be taught? Neurology. 2009;72(23):2020-3.
9. Dewey RB, Jr., Agostini M. Attitudes and performance of third- vs fourth-year neurology clerkship students. Arch Neurol. 2010;67(5):548-51.
10. Estevez ME, Lindgren KA, Bergethon PR. A novel three-dimensional tool for teaching human neuroanatomy. Anatomical sciences education. 2010;3(6):309-17.
11. Jensen R, Mitsikostas DD, Valade D, Antonaci F. Guidelines for the organization of headache education in Europe: the headache school II. J Headache Pain. 2010;11(2):161-5.
12. Whillier S, Lystad RP. The Effect of Face-to-Face Teaching on Student Knowledge and Satisfaction in an Undergraduate Neuroanatomy Course. 2010;6(4):239-45.
13. Kamel H, Dhaliwal G, Navi BB, Pease AR, Shah M, Dhand A, et al. A randomized trial of hypothesis-driven vs screening neurologic examination. Neurology. 2011;77(14):1395-400.
14. Mehr SE, Hassanzadeh G, Zahmatkesh M, Seyedian M, Arbabi M, Mirzazadeh A, et al. Medical students' viewpoint regarding the integrated module of basal ganglia. 2011.
15. Park JH, Son JY, Kim S, May W. Effect of feedback from standardized patients on medical students' performance and perceptions of the neurological examination. Medical teacher. 2011;33(12):1005-10.
16. Pearl PL, Pettiford JM, Combs SE, Heffron A, Healton S, Hovaguimian A, et al. Assessment of genetics knowledge and skills in medical students: insight for a clinical neurogenetics curriculum. Biochem Mol Biol Educ. 2011;39(3):191-5.
17. Reilly FD. Outcomes from Building System Courseware for Teaching and Testing in a Discipline-Based Human Structure Curriculum. 2011;4(4):190-4.
18. Safdieh JE, Lin AL, Aizer J, Marzuk PM, Grafstein B, Storey-Johnson C, et al. Standardized patient outcomes trial (SPOT) in neurology. Med Educ Online. 2011;16.
19. Tan NC, Kandiah N, Chan YH, Umapathi T, Lee SH, Tan K. A controlled study of team-based learning for undergraduate clinical neurology education. BMC Med Educ. 2011;11:91.
20. Holden KR, Cooper SL, Wong JG. Neuroscience curriculum changes and outcomes: medical university of South Carolina, 2006 to 2010. Neurologist. 2012;18(4):190-5.
21. Lukas RV, Adesoye T, Smith S, Blood A, Brorson JR. Student assessment by objective structured examination in a neurology clerkship. Neurology. 2012;79(7):681-5.
22. Moore FG, Chalk C. Improving the neurological exam skills of medical students. Can J Neurol Sci. 2012;39(1):83-6.
23. Drapkin ZA, Lindgren KA, Lopez MJ, Stabio ME. Development and Assessment of a New 3D Neuroanatomy Teaching Tool for MRI Training. 2013;8(6):502-9.
24. Ermak DM, Bower DW, Wood J, Sinz EH, Kothari MJ. Incorporating simulation technology into a neurology clerkship. J Am Osteopath Assoc. 2013;113(8):628-35.
25. Wiles CM. Introducing neurological examination for medical undergraduates--how I do it. Pract Neurol. 2013;13(1):49-50.
26. Magalhães LVB, Fernandes PT, Magalhães DSF, Bastos RR, Min LL. A Brazilian original pedagogical approach to the teaching of neurology. Arquivos de Neuro-Psiquiatria. 2014;72(10):747-52.
27. Merlin LR, Horak HA, Milligan TA, Kraakevik JA, Ali II. A competency-based longitudinal core curriculum in medical neuroscience. Neurology. 2014;83(5):456-62.
28. Albert DV, Yin H, Amidei C, Dixit KS, Brorson JR, Lukas RV. Structure of neuroscience clerkships in medical schools and matching in neuromedicine. Neurology. 2015;85(2):172-6.
29. Anwar K, Shaikh AA, Sajid MR, Cahusac P, Alarifi NA, Al Shedoukhy A. Tackling student neurophobia in neurosciences block with team-based learning. Med Educ Online. 2015;20:28461.
30. Blood AD, Park YS, Lukas RV, Brorson JR. Neurology objective structured clinical examination reliability using generalizability theory. Neurology. 2015;85(18):1623-9.
31. Dao V, Yeh PH, Vogel KS, Moore CM. Applied neuroanatomy elective to reinforce and promote engagement with neurosensory pathways using interactive and artistic activities. Anat Sci Educ. 2015;8(2):166-74.
32. Li AY-L, Carvalho H. Active Learning in Neuroscience: A Manipulative to Simulate Visual Field Defects. 2016;40(4):462-4.
33. Roze E, Flamand-Roze C, Meneret A, Ruiz M, Le Liepvre H, Duguet A, et al. 'The Move', an innovative simulation-based medical education program using roleplay to teach neurological semiology: Students' and teachers' perceptions. Rev Neurol (Paris). 2016;172(4-5):289-94.
34. Strowd RE, Salas RM, Cruz TE, Gamaldo CE. Neurology clerkship goals and their effect on learning and satisfaction. Neurology. 2016;86(7):684-91.
35. Alimoglu MK, Yardim S, Uysal H. The effectiveness of TBL with real patients in neurology education in terms of knowledge retention, in-class engagement, and learner reactions. Advances in physiology education. 2017;41(1):38-43.
36. Ansakorpi H, Sumelahti M-L, Kaasila R. Medical students' experience of emotions and success in neurological studies - What do they tell us? BMC medical education. 2017;17(1):68.
37. Brich J, Jost M, Brustle P, Giesler M, Rijntjes M. Teaching neurology to medical students with a simplified version of team-based learning. Neurology. 2017;89(6):616-22.
38. Ezeala-Adikaibe BA, Okpara T, Ekenze OS, Onodugo O, Ezeala-Adikaibe NP, Nnaji T, et al.

Knowledge of medical students about epilepsy: Need for a change. Nigerian journal of clinical practice. 2017;20(7):884-91.

1. Ong JJY, Chan YC. Medical Undergraduate Survey on Headache Education in Singapore: Knowledge, Perceptions, and Assessment of Unmet Needs. Headache. 2017;57(6):967-78.
2. Shiels L, Majmundar P, Zywot A, Sobotka J, Lau CSM, Jalonen TO. Medical student attitudes and educational interventions to prevent neurophobia: a longitudinal study. BMC Med Educ. 2017;17(1):225.
3. Hlavac RJ, Klaus R, Betts K, Smith SM, Stabio ME. Novel dissection of the central nervous system to bridge gross anatomy and neuroscience for an integrated medical curriculum. Anat Sci Educ. 2018;11(2):185-95.
4. Horak H, Englander R, Barratt D, Kraakevik J, Soni M, Tiryaki E, et al. Entrustable professional activities: A useful concept for neurology education. Neurology. 2018;90(7):326-32.
5. Lemmon ME, Gamaldo C, Salas RME, Saxena A, Cruz TE, Boss RD, et al. Education Research: Difficult conversations in neurology: Lessons learned from medical students. Neurology. 2018;90(2):93- 7.
6. Lewis A, Howard J, Watsula-Morley A, Gillespie C. An educational initiative to improve medical student awareness about brain death. Clinical Neurology and Neurosurgery. 2018;167:99-105.
7. Roze E, Worbe Y, Louapre C, Meneret A, Delorme C, McGovern E, et al. Miming neurological syndromes improves medical student's long-term retention and delayed recall of neurology. Journal of the neurological sciences. 2018;391:143-8.
8. Salas RME, Strowd RE, Ali I, Soni M, Schneider L, Safdieh J, et al. Incorporating sleep medicine content into medical school through neuroscience core curricula. Neurology. 2018;91(13):597- 610.
9. Shelley BP, Chacko TV, Nair BR. Preventing "Neurophobia": Remodeling Neurology Education for 21(st)-Century Medical Students through Effective Pedagogical Strategies for "Neurophilia". Ann Indian Acad Neurol. 2018;21(1):9-18.
10. Tan K, Chin HX, Yau CWL, Lim ECH, Samarasekera D, Ponnamperuma G, et al. Evaluating a bedside tool for neuroanatomical localization with extended-matching questions. Anat Sci Educ. 2018;11(3):262-9.
11. Bornkamm K, Steiert M, Rijntjes M, Brich J. A novel longitudinal framework aimed at improving the teaching of the neurologic examination. Neurology. 2019;93(24):1046-55.
12. Cumberland DM, Sawning S, Church-Nally M, Shaw MA, Branch E, LaFaver K. Experiential Learning: Transforming Theory into Practice through the Parkinson's Disease Buddy Program. Teaching and learning in medicine. 2019;31(4):453-65.
13. Grumer M, Brustle P, Lambeck J, Biller S, Brich J. Validation and perception of a key feature problem examination in neurology. PloS one. 2019;14(10):e0224131.
14. Henssen DJHA, van den Heuvel L, De Jong G, Vorstenbosch MATM, van Cappellen van Walsum A-M, Van den Hurk MM, et al. Neuroanatomy Learning: Augmented Reality vs. Cross- Sections. 2019;13(3):353-65.
15. Pokryszko-Dragan A, Mottershead J, Aitken G. Attitudes towards neurology among medical undergraduates. Neurologia i Neurochirurgia Polska. 2019;53(1):61-73.
16. Rezende AB, de Oliveira AGF, Vale TC, Teixeira LAS, Lima ARA, Lucchetti ALG, et al.

Comparison of Team-Based Learning versus Traditional Lectures in Neuroanatomy: Medical Student Knowledge and Satisfaction. 2019;13(5):591-601.

1. Biesalski AS, von Kirchbauer I, Schmidt-Graf F. Neurological teaching in times of crisis. GMS J Med Educ. 2020;37(7):Doc69.
2. Karamaroudis S, Poulogiannopoulou E, Sotiropoulos MG, Kalantzis T, Johnson EO. Implementing Change in Neuroanatomy Education: Organization, Evolution, and Assessment of a Near- Peer Teaching Program in an Undergraduate Medical School in Greece. 2020;13(6):694-706.
3. Keser Z, Rodriguez YA, Tremont J, Hsieh PH, McCullough LD, Sandrone S, et al. The role of residents in medical students' neurology education: current status and future perspectives. BMC Med Educ. 2020;20(1):115.
4. Kraakevik JA, Frederick M, Ryan N, Haedinger LA, Carney PA. An observational study of an approach to accommodate a fourth-year to third-year neurology clerkship curricular transition. Med Educ Online. 2020;25(1):1710331.
5. Moore FGA. A Diverse Specialty: What Students Teach Us About Neurology and "Neurophobia". Can J Neurol Sci. 2020;47(5):675-80.
6. Tsang ACO, Lee PP, Chen JY, Leung GKK. From bedside to webside: A neurological clinical teaching experience. Med Educ. 2020;54(7):660.
7. Curtis CM, Eubanks JE, Charles SC, Boyer PJ, Harrell KM, Markandaya M, et al. A Required, Combined Neurology-Physical Medicine and Rehabilitation Clerkship Addresses Clinical and Health Systems Knowledge Gaps for Fourth-Year Medical Students. Am J Phys Med Rehabil. 2021;100(2S Suppl 1):S17-S22.
8. Frey J, Neeley B, Umer A, Lewis JW, Lama A, Pawar G, et al. Training in Neurology: Neuro Day: An Innovative Curriculum Connecting Medical Students With Patients. Neurology. 2021;96(10):e1482-e6.
9. Gummerson CE, Lo BD, Porosnicu Rodriguez KA, Cosner ZL, Hardenbergh D, Bongiorno DM, et al. Broadening learning communities during COVID-19: developing a curricular framework for telemedicine education in neurology. BMC Med Educ. 2021;21(1):549.
10. Gummi R, Smith R, Govindarajan R. Expanding medical student interaction in neurology with a redesigned student interest group in neurology (sign) chapter. BMC medical education. 2021;21(1):217.
11. Hall S, Kurn O, Anbu D, Nagy E, Dean O, Robson A, et al. Introduction of the Modified Neuroanatomy Motivation Questionnaire and Its Role in Comparing Medical Student Attitudes Towards Learning Neuroanatomy Between Neuro-enthusiasts and Standard Students. Med Sci Educ. 2021;31(6):1823-30.
12. McGovern E, Louapre C, Cassereau J, Flamand-Roze C, Corsetti E, Jegatheesan P, et al.

NeuroQ: A neurophobia screening tool assesses how roleplay challenges neurophobia. J Neurol Sci. 2021;421:117320.

1. Merzougui WH, Myers MA, Hall S, Elmansouri A, Parker R, Robson AD, et al. Multiple-Choice versus Open-Ended Questions in Advanced Clinical Neuroanatomy: Using a National Neuroanatomy Assessment to Investigate Variability in Performance Using Different Question Types. 2021;14(3):296- 305.
2. Monteiro O, Bhaskar A, Wong IN, Ng AKM, Baptista-Hon DT. Teaching Bioelectricity and Neurophysiology to Medical Students Using LabAXON Simulations. 2021;45(4):702-8.
3. Mowchun JJ, Frew JR, Shoop GH. Education Research: A Qualitative Study on Student Perceptions of Neurology and Psychiatry Clerkship Integration. Neurology. 2021;96(3):e472-e7.
4. Ong KY, Gan ASP, Rajalingam P, Tan NCK, Tan K. Is Virtual Team-Based Learning Feasible and Effective in Teaching Neurolocalisation? Can J Neurol Sci. 2021:1-3.
5. Oster C, Farhood I, Klebe S, Kleinschnitz C, Peters L. Neurological examination course in an interactive webinar as a solution during a pandemic. An overview of the implementation, optimization as well as critical considerations. GMS journal for medical education. 2021;38(1):Doc9.
6. Pace A, Orr SL, Rosen NL, Safdieh JE, Cruz GB, Sprouse-Blum AS. The current state of headache medicine education in the United States and Canada: An observational, survey-based study of neurology clerkship directors and curriculum deans. Headache. 2021;61(6):854-62.
7. Pavão Martins I, Fonseca AC, Ferro JM. Undergraduate neurology teaching: Comparison of an inpatient versus outpatient clinical setting. European Journal of Neurology. 2021;28(7):e46-e7.
8. Sandrone S, Albert DV, Dunham SR, Kraker J, Noviawaty I, Palm M, et al. Training in Neurology: How Lessons Learned on Teaching, Well-being and Telemedicine During the COVID-19 Pandemic Can Shape the Future of Neurology Education. Neurology. 2021.
9. Shah VS, Allman A, Verbeck N, Quinn M, Prats MI. Ultrasound's Impact on Preclinical Medical Student Neurology Unit Grades: Findings After 2 Years. J Ultrasound Med. 2021;40(9):1903-10.
10. Togher Z, Fullam S, Callanan I, Kearney H, Tubridy N. An evaluation of optimal tutorial methodologies for neurology teaching at undergraduate level : Optimal tutorial methods for neurology. Ir J Med Sci. 2021;190(3):965-9.
11. Heitmann H, Wagner P, Fischer E, Gartmeier M, Schmidt-Graf F. Effectiveness of non-bedside teaching during the COVID-19 pandemic: a quasi-experimental study. BMC Med Educ. 2022;22(1):73.
12. Park JJ, Ooi SZY, Gillespie CS, Bandyopadhyay S, Chowdhury YA, Solomou G, et al. The Neurology and Neurosurgery Interest Group (NANSIG)-ten years of cultivating interest in clinical neurosciences. Acta Neurochir (Wien). 2022;164(4):937-46.
13. Rajan KK, Pandit AS. Comparing computer-assisted learning activities for learning clinical neuroscience: a randomized control trial. BMC Med Educ. 2022;22(1):522.
14. Toro J, Rivera JS, Rodriguez D, Serna LA, Gaitan J, Medina T, et al. A Simulated Hospital in a COVID-19 Pandemic Environment for Undergraduate Neurology Students. Neurology. 2022;98(18 SUPPL).
