# Supplementary figures and images for: Neurology Undergraduate Medical Education: A Scoping Review
Source: Eur J Neurol. 2025 Mar 13;32(3):e70061. doi: 10.1111/ene.70061 (PMC11904807; doi:10.1111/ene.70061)

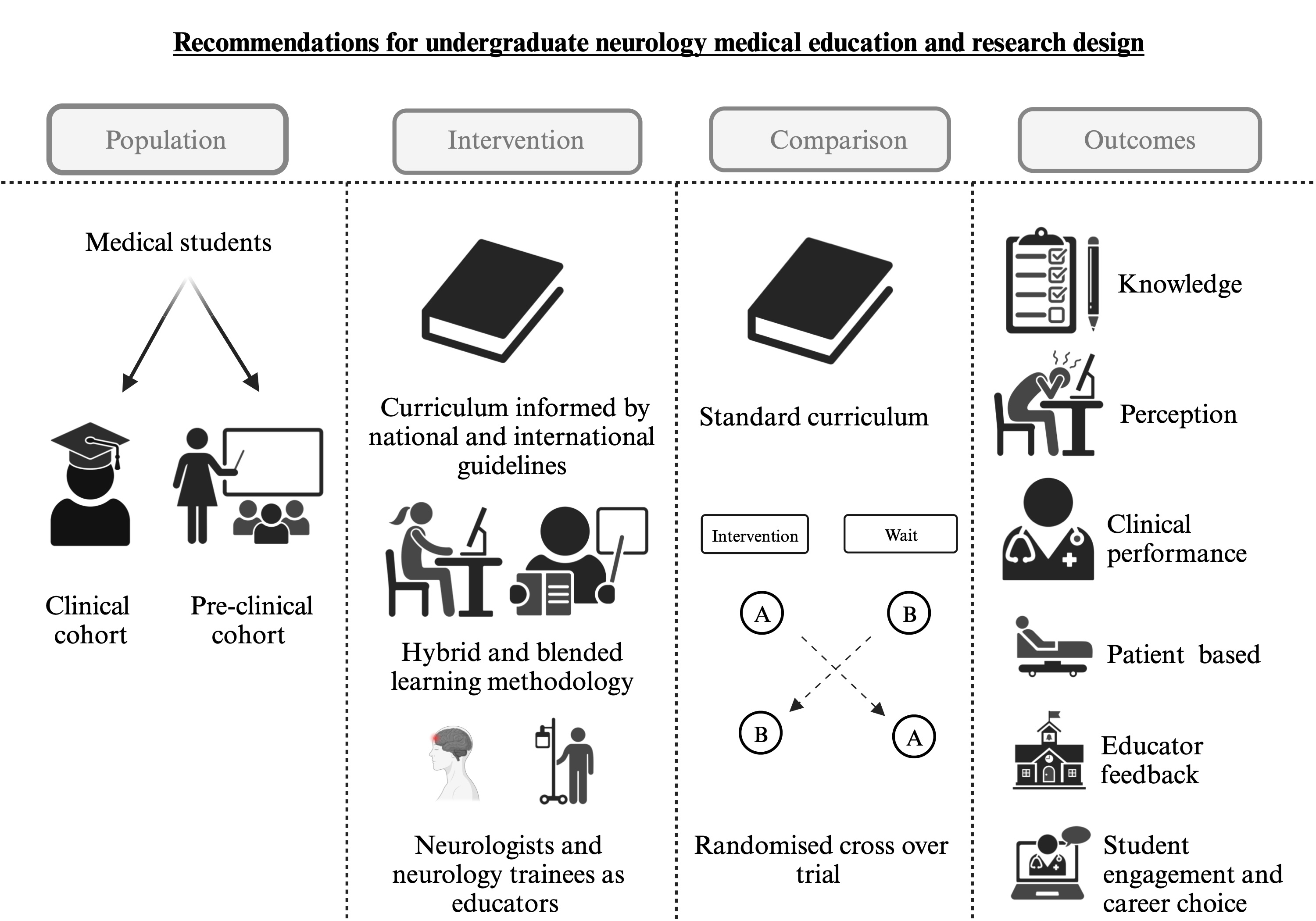

Supplement: Supplementary file 3 — Figure S1. [file ENE-32-e70061-s001.jpg]
